# TCEP-Enabled Click Modification of Glycidyl-Bearing Polymers with Biorelevant Sulfhydryl Molecules: Toward Chemoselective Bioconjugation Strategies

**DOI:** 10.1021/acs.biomac.5c00766

**Published:** 2025-07-17

**Authors:** Ilaria Porello, Federico Stucchi, Rosachiara Guarini, Giulia Sbaruffati, Francesco Cellesi

**Affiliations:** Department of Chemistry Materials and Chemical Engineering G. Natta, 18981 Politecnico di Milano, Via Luigi Mancinelli 7, Milan 20131, Italy

## Abstract

Thiol-epoxy ring opening is a highly efficient and versatile click reaction for postpolymerization modification, ideal for the conjugation of sulfhydryl-containing biomolecules. This study investigated the reactivity of thiols, disulfides, and amines toward glycidyl-bearing polymers, aiming to optimize thiol conjugation using tris­(2-carboxyethyl)­phosphine (TCEP) as a disulfide-reducing agent. Epoxide groups were introduced via glycidyl methacrylate (GMA) polymerized by ATRP to yield PGMA homopolymers and poly­(ε-caprolactone) (PCL)-based block copolymers. ^1^H NMR confirmed quantitative thiol functionalization, while amines showed poor reactivity. l-cysteine conjugation further demonstrated the reaction’s chemoselectivity. Thioglycerol conjugation yielded poly­(2-hydroxy-3-(thioglycerol)­propyl methacrylate) (PTGMA), a highly hydroxylated PEG alternative. Functionalization was extended to PCL-*b*-PGMA and PEGMA-based copolymers, forming amphiphilic nanoparticles via nanoprecipitation. Sequential modification with thioglycerol and the cRGD peptide yielded bioactive, size-controlled nanocarriers. Overall, a robust strategy has emerged for synthesizing multifunctional polymeric nanomaterials. Its compatibility with equimolar reactants under ambient conditions makes it particularly suited for the efficient incorporation of sensitive, high-value biomolecules into targeted drug delivery systems.

## Graphical abstract

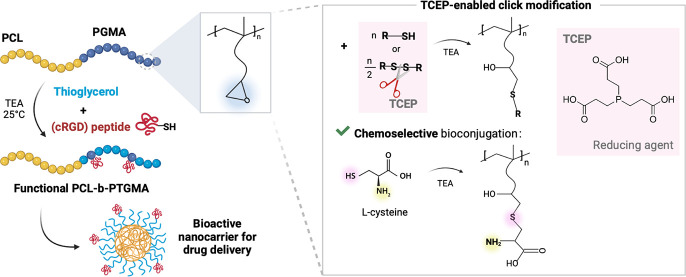

## 1 Introduction

The base-catalyzed oxirane ring-opening reaction is commonly adopted for the postpolymerization modification of polymers that present epoxide functional groups.^1,2^ The epoxide ring can react with nucleophiles such as amines, thiols, and alcohols, enabling diverse modifications and thus presenting functional versatility.^3–8^

In particular, the epoxide ring-opening process through thiol-nucleophiles represents an efficient route to functionalize synthetic polymers for biomedical applications.^9–14^ Such reactions are typically performed under ambient conditions and are well-known for their rapidity, efficiency, and regioselectivity^3,15–17^ (Figure S1). For these reasons, these transformations are frequently classified as click reactions as they ensure quantitative conversion, they proceed with minimal side reactions, and they can occur in aqueous or biocompatible solvents with excellent yield.^18–22^ The thiol-epoxy reaction is therefore suitable for the conjugation of sensitive peptides and proteins that present cysteine residues available for modification, preserving their biomolecular functionality for targeted therapy.^4,23–30^ Furthermore, it can be used for conjugation with a variety of commercially available thiols, providing the opportunity to extensively functionalize the polymer of interest and thereby impart the desired properties.^14,31–33^ This strategy provides several advantages over other thiol-based reactions, such as radical thiol–ene and Michael-type additions.^34,35^ It forms hydrolytically stable linkages and eliminates the need for radicals, offering better control and stability in polymer synthesis. Additionally, it avoids the use of reactive double bonds, thus reducing the risk of unwanted polymerization or cross-linking.

A common challenge in thiol-epoxy reactions is the tendency of free thiols to undergo oxidative dimerization under ambient conditions, resulting in the formation of disulfide bonds. These disulfides are unreactive under the basic conditions typically used in such transformations. As a result, an excess of thiol precursors is often needed to compensate for the stoichiometric imbalance caused by disulfide formation, thereby compromising the click reaction efficiency. This drawback can be overcome by regenerating free thiols using a reducing agent. Commonly used S–S bond reductants, such as dithiothreitol (DTT), are not suitable for the proposed reaction scheme, as they act through their thiolate form, which would interfere with the ring-opening reaction.^1,14^ Such undesired side reactions could compromise the molecular integrity and architecture of the modified polymers, affecting the resulting physicochemical behavior. Therefore, for this type of conjugation, it is essential to employ nonthiol-based reducing agents capable of selectively cleaving disulfide bonds without interfering with the desired functionalization process.^36^ For instance, sodium borohydride (NaBH_4_) has recently been proposed as an efficient reducing agent in thiol-epoxy conjugations,^1^ although caution is needed due to its high reactivity with water, which often requires an inert atmosphere to prevent moisture exposure.

In the thiol–ene click approach to polymer–protein conjugates, phosphines are commonly used as disulfide-reducing agents, providing quantitative substrate modification in a relatively short reaction time.^36,37^

Dimethylphenylphosphine (DMPP) and tris­(2-carboxyethyl)­phosphine) (TCEP) were examined in detail for conjugation in organic and water solvents, respectively.^38,39^ These compounds are relatively safe to handle and easy to remove for product isolation. TCEP is commonly favored over other reducing agents because of its potent reducing ability, excellent stability, broad pH tolerance, and lack of odor.^14,22,40–43^

Although phosphines are weaker nucleophiles toward epoxides compared to thiols and amines, their safe use as a chemoselective reducing agent in oxirane ring-opening reactions under click conditions should be investigated.

Moreover, a potential competition between thiols and amines for epoxide ring opening presents a significant selectivity challenge in achieving click conditions. This issue is particularly relevant when working with multifunctional molecules, such as peptides and proteins, where cysteine often coexists with lysine residues at the N-terminus. Since thiols are generally stronger nucleophiles than amines, the opening of epoxide rings by amines typically requires longer reaction times and higher temperatures.^3,6,7,32,44^ Therefore, identifying optimal reaction conditions is crucial to maximize the chemoselectivity toward thiols and to obtain the target conjugation product.

This study investigated and compared the efficiency of different reagents (thiols, disulfides, and amines) in the thio-epoxy click modification of polymers. The goal was to establish optimal conditions for polymer conjugation with thiols using phosphine-based reducing agents, particularly TCEP (Figure 1).

**Figure 1:**
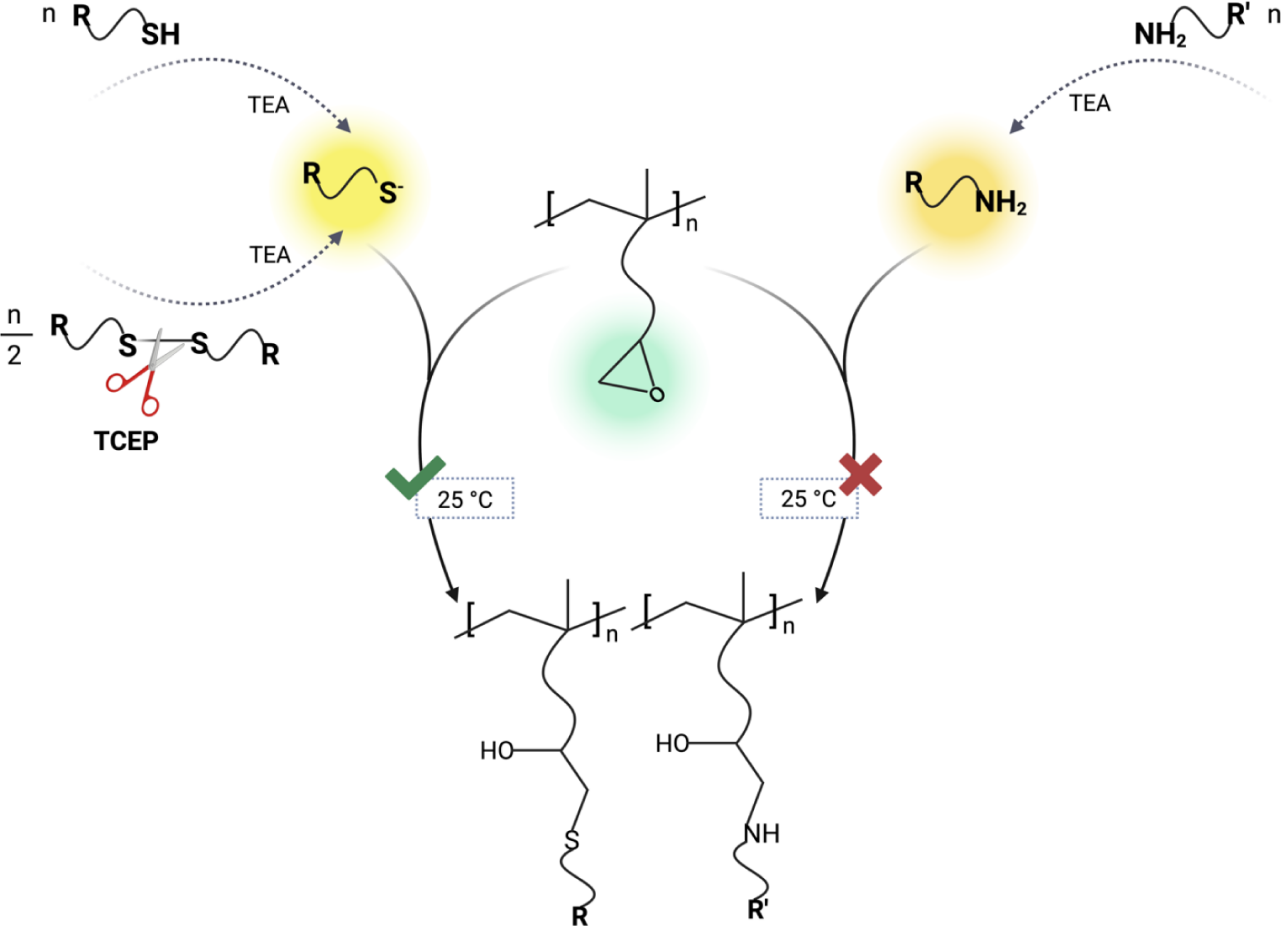
Comparative analysis of reaction pathways for glycidyl modification via click ring-opening reactions with thiols, disulfides, and amines. While thiols and disulfides induce efficient functionalization, amines do not react effectively under the same mild conditions.

Furthermore, we explored whether the thio-epoxy reaction can serve as a powerful alternative to thiol–ene reactions for bioconjugation and polymer postfunctionalization under mild conditions, enabling the synthesis of functional macromolecules tailored for biomedical applications.^45^

In order to carry out the comparative study, epoxide side groups were introduced into the tested polymers by using glycidyl methacrylate (GMA) as a monomeric unit. GMA can be polymerized via well-established Reversible-Deactivation Radical Polymerization (RDRP) techniques, such as Reversible Addition–Fragmentation Chain Transfer (RAFT) polymerization, Nitroxide-Mediated polymerization (NMP) and various forms of Atom Transfer Radical Polymerization (ATRP),^3,4,46–48^ as Activators Regenerated by Electron Transfer (ARGET) ATRP, Initiators for Continuous Activator Regeneration (ICAR) ATRP, or electrochemically mediated ATRP (eATRP).^49,50^ This versatile monomer enables the synthesis of PGMA homopolymers or can be employed as a comonomer for the design of more complex polymer architectures with tailored topologies, due to its reactive functionality.^40,41,51,52^

PGMA homopolymers with a predetermined degree of polymerization, synthesized via ATRP, were initially treated with a library of selected thiols, disulfides, and amines under various reaction conditions.

After a systematic investigation of the reactivity of these compounds with the epoxy groups of PGMA, the study was extended to more complex molecular architectures. Specifically, ATRP macroinitiators based on poly­(ε-caprolactone) (PCL) were copolymerized with GMA and PEGMA. Once functionalized via a thio-epoxy reaction, the resulting biocompatible amphiphilic copolymers were used to formulate nanoparticle (NP) suspensions in aqueous buffer, forming a core–shell structure where PCL constitutes the hydrophobic core. The ability to integrate the physicochemical properties of these copolymers with thio-epoxy postfunctionalization was evaluated to achieve multifunctional NPs with tailored properties, which offer strong potential for targeted drug delivery applications.

## 2 Materials and Methods

### 2.1 Materials

ε-Caprolactone 97% (ε-CL), Glycidyl methacrylate 97% (GMA), Poly­(ethylene glycol) methyl ether methacrylate *M*_n_ = 500 Da (PEGMA), α-Bromoisobutyryl bromide 98% (BiBB), Ethyl-α-bromoisobutyrate 98% (EBiB), 2,2’-Bipyridyl ≥ 99% (BPY), Copper­(I) bromide 98% (CuBr­(I)), Tin­(II) 2-ethylhexanoate 92–100% (Sn­(Oct)_2_), Deuterochloroform ≥ 99.8% (CDCl_3_), Aluminum oxide (Al_2_O_3_), Sodium bicarbonate (NaHCO_3_), Sodium sulfate ≥ 99% (Na_2_SO_4_), Sodium chloride ≥ 99% (NaCl), Dichloromethane ≥ 99.8% (DCM), Methanol ≥ 99.8% (MeOH), *N*,*N*-Dimethylformamide ≥ 99.8% (DMF), Diethyl ether ≥ 99.8% (Et_2_O), Hexane ≥ 99.8%, *N*,*N*-Dimethylformamide ≥ 99.8% anhydrous (DMF), Tetrahydrofuran ≥ 99.9% anhydrous inhibitor-free (THF), Tetrahydrofuran ≥ 99.9% containing 250 ppm BHT as inhibitor (THF), Toluene ≥ 99.9% anhydrous, Thioacetic acid 96% (TAA), Thiophenol ≥ 99%, 1-Thioglycerol ≥ 99% (GC), Phenyl disulfide ≥ 99%, 2-Hydroxyethyl disulfide technical grade, l-Cysteine ≥ 97% (FG), Glycine ACS Reagent ≥ 98.5%, Benzylamine ReagentPlus 99%, Ethanolamine ≥ 98%, Propargylamine 98%, Allylamine hydrochloride 98%, Triethylamine ≥ 99% (TEA), 4-(Dimethylamino)­pyridine ≥ 99% (DMAP), Acetyl chloride ≥ 99% (CH_3_COCl), 5,5′-Dithio-bis­(2-nitrobenzoic acid) ≥ 98% (DTNB, Ellman’s Reagent), Tris­(2-carboxyethyl)­phosphine 98% (TCEP), Tris­(2-carboxyethyl)­phosphine immobilized on Agarose CL-4B (TCEP) were all purchased from Sigma-Aldrich (Merck, Italy). Dimethyl sulfoxide-d_6_ 100% (DMSO-*d*_6_) and deuterium oxide 100% (D_2_O) were purchased from Eurisotop (Cambridge Isotope Laboratories, France). The cyclic RGD peptide (cRGD) (Arg-Gly-Asp-(d-Phe)-Cys, purity 98.03%, *M*_n_ = 580.6 Da) was synthesized and purchased from CASLO (ApS, Technical University of Denmark, DTU). All chemicals were used without further purification, unless otherwise indicated. Deionized water (18.2 MΩ) was obtained from a Millipore Milli-Q purification unit.

### 2.2 Polymer Synthesis and Characterization

The extent of monomer conversion (χ_ROP_ or χ_ATRP_), the degree of polymer functionalization (χ_Funct._), and the polymer’s degree of polymerization (DP) were quantified via ^1^H NMR spectroscopy. Crude and purified polymers were dissolved in deuterated solvents (CDCl_3_, DMSO-*d*_6_, or D_2_O), and spectra were recorded on a Bruker Avance 400 MHz NMR at 298 K. Chemical shifts (δ) are reported in ppm, referenced to the solvent’s residual signal.

Polymer number-average molar mass (*M*_n,SEC_), weight-average molar mass (*M*_w,SEC_), and dispersity (*Đ* = *M*_w_/*M*_n_) were assessed by size exclusion chromatography (SEC), utilizing a Jasco LC-2000Plus system. The setup included a PU-2080 pump, a CO-2060Plus column oven, a RI-2031Plus refractive index detector, and an autosampler (Jasco AS-2055Plus). The system was equipped with three Agilent PLgel columns (300 × 7.5 mm, MW range: 5 × 10^2^ to 1.7 × 10^6^ g/mol, 5 μm particle size) preceded by a PLgel guard column (50 × 7.5 mm, 5 μm particle size). THF was used as an eluent at 35 °C, with a flow rate of 1 mL/min. Samples were dissolved in THF (4 mg/mL, containing 250 ppm of BHT) and filtered through PTFE syringe filters (0.22 μm). Calibration was performed using polystyrene standards from RESTEK and Sigma-Fluka.

#### 2.2.1 Synthesis of PGMA_n_

PGMA_n_ homopolymer was synthesized following a protocol adapted from previously reported methods.^48^ EBiB was selected as the reaction initiator, using a GMA/initiator molar ratio of *n*, where *n* = 30. Full polymerization details are provided in the Supporting Information.

Yield >90%. ^1^H NMR (400 MHz, DMSO-*d*_6_), δ (ppm): δ 4.29, 3.75 (s, 2H·*n*, −C­(O)–O–C*H*_2_–(CH–CH_2_–O)), 3.21 (s, 1H·*n*, −C­(O)–O–CH_2_–(C*H*–CH_2_–O)), 2.80, 2.66 (s, 2H·*n*, −C­(O)–O–CH_2_–(CH–C*H*_2_-O)), 2.06–1.70 (m, 2H·*n*, Backbone: −C*H*_2_–C­(CH_3_)­(O)−), 1.05–0.59 (m, 3H·*n*, Backbone: −CH_2_–C­(C*H*_3_)­(O)−), where *n* is the PGMA degree of polymerization.

#### 2.2.2 Synthesis of PCL_r_ **-** *b* **-**PGMA_n_

PCL_r_-PGMA_n_ block copolymer was synthesized according to a previously reported protocol.^48^ The ratio between the macroinitiator (*I*) PCL_r_-Br and the monomer GMA was set as follows: eq GMA/eq *I* = *n*, where *n* = 30. Full polymerization details are provided in the Supporting Information.

Yield 90%. ^1^H NMR (400 MHz, CDCl_3_), δ (ppm): δ 4.30, 3.75 (s, 2H·*n*, PGMA: −C­(O)–O–C*H*_2_–(CH­(O)–CH_2_)), 3.98 (t, 2H·(*r*–1), PCL: −O–C­(O)–(CH_2_)_4_–C*H*_2_−), 3.20 (s, 1H·*n*, PGMA: −C­(O)–O–CH_2_–(C*H*(O)–CH_2_)), 2.80, 2.66 (s, 2H·*n*, −C­(O)–O–CH_2_–(CH­(O)–C*H*_2_)), 2.27 (t, 2H·*r*, PCL: −O–C­(O)–C*H*_2_–(CH_2_)_4_−), 2.04–1.69 (m, 2H·*n*, Backbone: −C*H*_2_–C­(CH_3_)­(O)−), 1.61–1.48 (m, 4H·*r*, PCL: −O–C­(O)–CH_2_–C*H*_2_–CH_2_–C*H*_2_-CH_2_−), 1.36–1.25 (m, 2H·*r*, PCL: −O–C­(O)–(CH_2_)_2_–C*H*_2_–(CH_2_)_2_−), 1.08–0.71 (m, 3H·*n*, Backbone: −CH_2_–C­(C*H*_3_)­(O)−), where *n* is the PGMA degree of polymerization, and *r* is the PCL degree of polymerization.

#### 2.2.3 Synthesis of PCL_r_ **-** *b* **-**[P­(PEGMA)_m_-*co*-PGMA_n_]

PCL_r_-*b*-[P­(PEGMA)_m_-*co*-PGMA_n_] block copolymer was synthesized according to a previously reported protocol.^48^ The ratio between the macroinitiator (*I*) PCL_r_-Br and the monomers GMA and PEGMA was set as follows: eq PEGMA/eq *I* = *m*, eq GMA/eq *I* = *n*, where *m* = 30 and *n* = 3, 4, and 5. Full polymerization details are provided in the Supporting Information.

Yield 80%. ^1^H NMR (400 MHz, CDCl_3_), δ (ppm): δ 4.28, 3.82 (s, 2H·*n*, PGMA: −C­(O)–O–C*H*_2_–(CH­(O)–CH_2_)), 4.16–4.00 (m, 2H·(*r*–1) + 2H·*m*, PCL: −O–C­(O)–(CH_2_)_4_–C*H*_2_–, P­(PEGMA): −C­(O)–O–C*H*_2_–CH_2_−), 3.74 (t, 2H·*m*, P­(PEGMA): −C­(O)–O–CH_2_–C*H*_2_-), 3.70–3.59 (m, 30H·*m*, P­(PEGMA): −O–((C*H*_2_)_2_–O)_7_–C*H*_2_–CH_2_–O–CH_3_), 3.55 (t, 2H·*m*, P­(PEGMA): −CH_2_–C*H*_2_–O–CH_3_), 3.38 (s, 3H·*m*, P­(PEGMA): −CH_2_–O–C*H*_3_), 3.20 (s, 1H·*n*, PGMA: −C­(O)–O–CH_2_–(C*H*(O)–CH_2_)), 2.84, 2.64 (s, 2H·*n*, −C­(O)–O–CH_2_–(CH­(O)–C*H*_2_)), 2.30 (t, 2H·*r*, PCL: −O–C­(O)–C*H*_2_–(CH_2_)_4_−), 2.08–1.73 (m, 2H·(*n* + *m*), P­(PEGMA) and PGMA: Backbone: −C*H*_2_–C­(CH_3_)­(O)−), 1.67–1.60 (m, 4H·*r,* PCL: −O–C­(O)–CH_2_–C*H*_2_–CH_2_–C*H*_2_-CH_2_−), 1.43–1.34 (m, 2H·*r*, PCL: −O–C­(O)–(CH_2_)_2_–C*H*_2_–(CH_2_)_2_−), 1.09–0.79 (m, 3H·(*n* + *m*), P­(PEGMA) and PGMA: Backbone: −CH_2_–C­(C*H*_3_)­(O)−), where *n* is the PGMA degree of polymerization, *r* is the PCL degree of polymerization, and *m* is the P­(PEGMA) degree of polymerization.

### 2.3 PGMA Postpolymerization Modification

The reaction and purification conditions (molar ratios, reagents, solvents, temperatures) reported for the functionalization of GMA with thiolated molecules and amines are to be considered valid both for PGMA_n_ homopolymers and for copolymers containing a PGMA_n_ segment or a random block of P­(PEGMA)_m_ and PGMA_n_.

### 2.4 PGMA_n_ Functionalization with Thiophenol/Thioacetic Acid/Thioglycerol

THF (inhibitor-free) or DMF were degassed under N_2_ for 30 min. 500 mg of polymer containing glycidyl units was added in a Schlenk flask, and three cycles of N_2_/vacuum were performed. 5 mL of degassed THF or DMF were used to dissolve the polymer under stirring with continuous N_2_ flux. The solvent was selected based on the solubility of the considered functionalizing agent. Specifically, DMF was employed for functionalization with thioglycerol, and both solvents were tested in the case of thiophenol and thioacetic acid. Thiophenol/thioacetic acid (TAA)/thioglycerol and TEA were then added to the reaction mixture, maintaining the system under nitrogen flux. The selected amounts of TEA and thiophenol/thioacetic acid/thioglycerol were varied in different tests to determine how the reaction conditions affected the resulting conversion. The complete list of the reagent molar ratios tested is reported in Table 2. After being stirred overnight at 25 °C, the reaction mixture was evaporated under vacuum, and the resulting crude material was redissolved in DCM (2.5 mg/mL). In the case of thiophenol functionalization, the polymer solution was dropped in cold MeOH (DCM/MeOH = 1/100 v/v) while in the presence of thioglycerol functionalization, the polymer solution was dropped in cold Et_2_O (DCM/Et_2_O = 1/100 v/v), continuously stirring the system in an ice bath. In both cases, the mixtures were then cooled to −20 °C for 30 min, and the precipitate was recovered by decanting the supernatant via filtration. Alternatively, for the purification of polymer functionalized with thioacetic acid, the crude material was dissolved in 15 mL of DCM (φ_org_). A saturated aqueous NaCl solution (φ_acq_) was prepared. The polymer solution (φ_org_) was transferred in a separating funnel and washed three times with the NaCl solution (φ_org_/φ_acq_ ∼ 4/1 v/v for each wash). The aqueous phase collected after the washes was then extracted with additional DCM to ensure complete recovery of the product. Anhydrous Na_2_SO_6_ was added to the organic solution to remove any residual water, and the solution was filtered to remove salts. The filtrate was evaporated under reduced pressure, redissolved in 2 mL of DCM, and added dropwise to 250 mL of hexane, maintaining stirring throughout the process. The precipitate was recovered by decanting the supernatant via filtration

In the case of cofunctionalization where *X*% of a PGMA chain was selectively functionalized with the functionalizing agent *A* and its *Y*% with a different agent *B*, the reported reaction conditions remained unchanged. However, the following molar ratios were applied: *A*/eq epoxy ring = *X*/100/1 for the first step, and eq *B*/eq epoxy ring = *Y*/100/1 for the second step. At the end of each functionalization stage, if necessary, an aliquot of the product was purified as previously described and analyzed by ^1^H NMR to verify the conversion. Subsequently, the same product was subjected to the second functionalization step following the procedure specific to the functionalizing agent in question, along with the corresponding purification.

#### 2.4.1 Thiophenol Functionalization

Yield >80%. ^1^H NMR (400 MHz, DMSO-*d*_6_), δ (ppm): δ 7.41–7.05 (m, 5H·*n*, −CH_2_–S–C_6_ *H*_5_), 5.30 (s, 1H·*n*, −CH­(O*H*)–CH_2_–S–C_6_H_5_), 4.07–3.75 (s, 3H·*n*, −C­(O)–O–C*H*_2_–C*H*(OH)–CH_2_–S−), 3.13–2.89 (m, 2H·*n*, −CH­(OH)–C*H*_2_–S–C_6_H_5_), 2.05–1.57 (m, 2H·*n*, Backbone: −C*H*_2_–C­(CH_3_)­(O)−), 1.04–0.47 (m, 3H·*n*, Backbone: −CH_2_–C­(C*H*_3_)­(O)−) where *n* is the PGMA degree of polymerization.

#### 2.4.2 Thioacetic Acid Functionalization

Yield >80%. ^1^H NMR (400 MHz, DMSO-*d*_6_), δ (ppm): 5.40–4.9 (m, 1H·*n*, −CH­(O*H*)–CH_2_–S–CH_2_), 4.50–3.59 (m, 3H·*n*, −O– C*H*_2_–CH­(OH)–CH_2_–S−), 2.36 (s, 2H·*n*, −CH­(OH)–C*H*_2_–S−), 2.14–1.94 (m, 3H·*n*, −CH_2_–S–C­(O)–C*H*_3_), 1.95–1.58 (m, 2H·*n*, Backbone: C*H*_2_–C­(CH_3_)­(C­(O))−), 1.03–0.5 (m, 3H·*n*, Backbone: CH_2_–C­(C*H*_3_)­(C­(O))−) where *n* is the PGMA degree of polymerization.

#### 2.4.3 Thioglycerol Functionalization

Yield >80%. ^1^H NMR (400 MHz, DMSO-*d*_6_), δ (ppm): 5.11 (s, 1H·*n*, −CH­(O*H*)–CH_2_–S–CH_2_), 4.77, 4.55 (s, 2H·*n*, −S–CH_2_–CH­(O*H*)–CH_2_–O*H*), 4.06–3.72 (m, 3H·*n*, −O–C*H*_2_–C*H*(OH)–CH_2_–S−), 3.6 (s, 1H·*n*, −CH_2_–C*H*(OH)–CH_2_–OH), 2.79–2.54 (m, 3H·*n*, −CH­(OH)–CH_2_–S–CH_2_–CH­(OH)–C*H*_2_–OH), 1.03–0.5 (m, 3H·*n*, Backbone: CH_2_–C­(C*H*_3_)­(C­(O))−) where *n* is the PGMA degree of polymerization.

### 2.5 PGMA_n_ Functionalization with Benzylamine/Propargylamine/Glycine

DMF was degassed under N_2_ for 30 min. 500 mg of polymer-bearing glycidyl units was added in a Schlenk flask, and three cycles of N_2_/vacuum were performed. Five mL of degassed DMF were used to dissolve the polymer under stirring with continuous N_2_ flux. Benzylamine/propargylamine/glycine and TEA were then added to the reaction mixture, maintaining the N_2_ flux. The selected amount of TEA and benzylamine/propargylamine/glycine were varied in different tests to determine how the reaction conditions affected the resulting conversion. The complete list of the reagents’ molar ratios tested is reported in Table 2. The solution was stirred overnight at either 25 or 60 °C to study the effect of temperature on functionalization performance. The reaction mixture was subsequently evaporated under a vacuum, and the resulting crude material was redissolved in DCM (2.5 mg/mL). In the case of benzylamine and glycine functionalization, the polymer solution was dropped in cold MeOH (DCM/MeOH = 1/100 v/v), while when propargylamine functionalization was performed, the polymer solution was dropped in cold Et_2_O (DCM/Et_2_O = 1/100 v/v) continuously stirring the system in an ice bath. The mixtures were then cooled to −20 °C for 30 min, and the precipitate was recovered by decanting the supernatant via filtration.

#### 2.5.1 PGMA_n_ Functionalization with Diphenyl Disulfide/2-Hydroxyethyl Disulfide

THF (inhibitor-free) or DMF were degassed under N_2_ flux for 30 min. 500 mg of polymer-bearing glycidyl units was added in a Schlenk flask, and three cycles of N_2_/vacuum were performed. Five mL of degassed THF or DMF were used to dissolve the polymer under stirring with continuous N_2_ flux. The solvent was selected based on the solubility of the considered functionalizing agent. Specifically, THF and DMF were used for functionalization with diphenyl disulfide, while DMF was used for modification employing 2-hydroxyethyl disulfide. Diphenyl disulfide/2-hydroxyethyl disulfide and the reducing agent TCEP (eq diphenyl disulfide/eq TCEP = 1/0.5 or eq 2-hydroxyethyl disulfide/eq TCEP = 1/0.5) were dissolved in 1 mL of degassed DMF and stirred for 15 min. The diphenyl disulfide-TCEP solution/2-hydroxyethyl disulfide-TCEP solution and TEA were then added to the reaction mixture, maintaining the system under nitrogen flux. Considering diphenyl disulfide modification, the selected amount of TEA was varied in different tests to determine the minimum volume of catalyst required to reach complete conversion (eq epoxy ring/eq diphenyl disulfide/eq TEA = 1/0.5/3, eq epoxy ring/eq diphenyl disulfide/eq TEA = 1/0.5/1). To better understand the effect of TCEP, tests were also carried out running the reaction in the same condition aforementioned but avoiding the diphenyl disulfide pretreatment with the reducing agent, directly inserting it in the reaction flask with TEA in both THF and DMF. The kinetic profile of the diphenyl disulfide functionalization in THF or DMF, both in the presence of TCEP and in its absence, was monitored by withdrawing aliquots of the reaction mixture at specific time points (1, 2, 3, 4, 6, and 18 h) to perform ^1^H NMR analysis. Concerning 2-hydroxyethyl disulfide functionalization, the following molar ratios were employed: eq epoxy ring/eq 2-hydroxyethyl disulfide/eq TEA = 1/0.5/3. After stirring overnight at 25 °C, the reaction mixtures were evaporated under vacuum, and the resulting crude material was redissolved in DCM (2.5 mg/mL) and dropped in cold MeOH (DCM/MeOH = 1/100 v/v) in the case of diphenyl disulfide functionalization, or in cold H_2_O (DCM/H_2_O = 1/100 v/v) in the case of 2-hydroxyethyl disulfide functionalization. The mixtures were then cooled to −20 °C for 30 min, and the precipitate was recovered by decanting the supernatant via filtration.

#### 2.5.2 Diphenyl Disulfide Functionalization

Yield >80%. ^1^H NMR (400 MHz, DMSO-*d*_6_), δ (ppm): δ 7.41–7.05 (m, 5H·*n*, −CH_2_–S–C_6_ *H*_5_), 5.30 (s, 1H·*n*, −CH­(O*H*)–CH_2_–S–C_6_H_5_), 4.07–3.75 (s, 3H·*n*, −C­(O)–O–C*H*_2_–C*H*(OH)–CH_2_–S−), 3.13–2.89 (m, 2H·*n*, −CH­(OH)–C*H*_2_–S–C_6_H_5_), 2.05–1.57 (m, 2H·*n*, Backbone: −C*H*_2_–C­(CH_3_)­(O)−), 1.04–0.47 (m, 3H·*n*, Backbone: −CH_2_–C­(C*H*_3_)­(O)−) where *n* is the PGMA degree of polymerization.

#### 2.5.3 2-Hydroxyethyl Disulfide Functionalization

Yield >80%. ^1^H NMR (400 MHz, DMSO-*d*_6_), δ (ppm): 5.11 (s, 1H·*n*, −CH­(O*H*)–CH_2_–S–CH_2_), 4.77 (s, 1H·*n*, −S–CH_2_–CH_2_–O*H*), 4.06–3.72 (m, 3H·*n*, −O–C*H*_2_–C*H*(OH)–CH_2_–S−), 3.6 (s, 2H·*n*, −S–CH_2_–C*H*_2_–OH), 2.75–2.54 (m, 4H·*n*, −CH­(OH)–C*H*_2_–S–C*H*_2_–CH_2_–OH), 2.10–1.58 (m, 2H·*n*, Backbone: −C*H*_2_-C­(CH_3_)­(O)−), 1.03–0.5 (m, 3H·*n*, Backbone: CH_2_–C­(C*H*_3_)­(C­(O))−) where *n* is the PGMA degree of polymerization.

### 2.6 PGMA_n_ Functionalization with l-Cysteine

DMF was degassed under N_2_ for 30 min. 50 mg of polymer-bearing glycidyl units were added in a Schlenk flask, and three cycles of N_2_/vacuum were performed. Five mL of degassed THF or DMF were used to dissolve the polymer under stirring with continuous N_2_ flux. l-Cysteine (l-Cys) and the reducing agent TCEP (eq l-Cys/eq TCEP = 1/0.5) were dissolved in 1 mL of DMF and stirred for 15 min. l-Cys/TCEP solution and TEA were then added to the reaction system (eq epoxy ring/eq l-Cys/eq TEA = 1/1/1) under N_2_ flux. After being stirred overnight at 25 °C, the reaction mixtures were evaporated under vacuum, and the resulting crude material was redissolved in DCM (2.5 mg/mL) and dropped in cold Et_2_O (DCM/Et_2_O = 1/100 v/v) keeping the system in an ice bath. The mixtures were then cooled to −20 °C for 30 min, and the precipitate was recovered by decanting the supernatant via filtration. Yield >80%. ^1^H NMR (400 MHz, DMSO-*d*_6_), δ (ppm): 5.11 (s, 1H·*n*, −CH_2_–CH­(O*H*)–CH_2_–S−), 4.8–4.35 (m, 3H·*n*, −S–CH_2_–CH­(N*H*_2_)–C­(O)–O*H*), 4.06–3.72 (m, 3H·*n*, −O–C*H*_2_–C*H*(OH)–CH_2_–S−), 3.6 (s, 1H·*n*, −S–CH_2_–C*H*(NH_2_)–C­(O)–OH), 2.8–2.5 (m, 4H·*n*, −CH­(OH)–C*H*_2_–S–C*H*_2_–CH­(H_2_N)−), 2.06–1.62 (m, 2H·*n*, Backbone: −C*H*_2_–C­(CH_3_)­(O)−), 1.11–0.6 (m, 3H·*n*, Backbone: CH_2_–C­(C*H*_3_)­(C­(O))−) where *n* is the PGMA degree of polymerization.

To verify the presence of free −SH groups after l-cysteine attachment to glycidyl scaffolds, an aliquot of the resulting product was processed according to the Ellman’s Reagent (DTNB) assay procedure for quantitating sulfhydryl groups based on molar absorptivity (Thermo Scientific, Instructions Ellman’s Reagent, 22582).

The quantification of free sulfhydryl groups was carried out by correlating the absorbance (*A*) of the sample with the thiol molar concentration (*c*), based on the known molar extinction coefficient (*E*) of TNB at 412 nm. UV–Vis measurements were performed at 25 °C using a Jasco V-630 spectrophotometer employing 1 cm path length (*b*) disposable PMMA cuvettes. Under these conditions, the molar absorptivity of TNB was considered equal to *E* = *A*/*bc* = 14 150 cm^–1^ M^–1^ according to the Beer–Lambert law.

Before the described analysis, the modified polymer was processed using an immobilized TCEP disulfide reducing gel (Thermo Scientific, Instructions for Immobilized TCEP Disulfide Reducing Gel, 77712) in batch format, following the manufacturer’s protocol for sample volumes between 20 and 750 μL. The actual concentration of free thiol groups in the polymer was evaluated after accounting for the dilution factors introduced during sample preparation.

#### 2.6.1 Acetylation of l-Cysteine-Modified PGMA_n_

Cysteine-modified PGMA_n_ (70 mg, containing 0.27 mmol of hydroxyl groups and 0.27 mmol of primary amines) was dissolved in anhydrous DMF (2 mL) and stirred at r.t. until complete dissolution. TEA (0.15 mL, 1.09 mmol) and DMAP (6.6 mg, 0.05 mmol) were separately dissolved in 0.5 mL of DMF and subsequently added to the polymer solution. Acetyl chloride (0.08 mL, 1.09 mmol), previously dissolved in 0.5 mL of DMF, was then added dropwise to the reaction mixture at 0 °C under stirring. The reaction was allowed to proceed at r.t. for 18 h. Upon completion, DMF and low-molecular-weight byproducts were removed by dialysis against Milli-Q water (3.5 kDa MWCO dialysis membrane). The precipitated polymer was dissolved in DCM (2.5 mL), precipitated into cold hexane (DCM/hexane = 1/100 v/v), and collected by filtration followed by drying at reduced pressure.

#### 2.7 PGMA_n_ Functionalization with cRGD Peptide

DMF was degassed under N_2_ flux for 30 min. 50 mg of copolymer-bearing glycidyl units were added in a Schlenk flask, and three cycles of N_2_/vacuum were performed. Two mL of DMF were employed to dissolve the polymer under N_2_ flux. The cRGD peptide and the reducing agent TCEP (eq cRGD/eq TCEP = 1/0.5) were dissolved in 1 mL of DMF and stirred for 15 min. The cRGD/TCEP solution and TEA were then inserted into the reaction mixture (eq epoxy ring/eq cRGD/eq TEA = 1/0.1/3) preserving the N_2_ flux. The solution was stirred overnight at 25 °C. The resulting mixture was subsequently treated for functionalizing the remaining GMA units with thioglycerol by employing the procedure previously described. The crude obtained was dried under reduced pressure and redispersed in 1.5 mL of ultrapure water. Residual unreacted components were removed by ultrafiltration operated at 2500 RCF for 10 min using centrifugal filter units (Amicon, MWCO 10 kDa). The retentate was washed four times with 1 mL of PBS. The isolated product was then freeze-dried. Yield >70%.

### 2.8 Nanoparticle Formulation

NPs were obtained via a nanoprecipitation technique coupled with a solvent evaporation. Specifically, 10 mg of PCL_r_-*b*-PGMA_n_ functionalized either with thioglycerol alone or in combination with cRGD peptide were dissolved with 1 mL of acetone. The resulting organic phase was slowly added dropwise into 1 mL of PBS (10 mM, pH 7.4). The organic solvent was eliminated using a rotary evaporator at 32 °C under a controlled pressure gradient, starting from 1000 mbar down to 100 mbar over 1 h, followed by an additional hour at 100 mbar. A 700 μL aliquot was collected from each nanoparticle batch for subsequent analysis. Hydrodynamic diameter, polydispersity index (PdI), and particle size distribution were measured at 25 °C using Dynamic Light Scattering (DLS) on a Malvern Zetasizer Nano ZS instrument equipped with a 4  mW He–Ne laser (λ = 634 nm) and operating at a backscattering angle of 173°.

## 3 Results and Discussion

### 3.1 Polymer Design and Characterization

The PGMA_n_ homopolymers were synthesized via ATRP in THF starting from the GMA monomer, using EBiB as the reaction initiator and Cu­(I)Br as the catalyst, with TPMA as the ligand. These homopolymers served as the initial platform for evaluating the postpolymerization modification of glycidyl epoxy rings with various functionalizing agents. The strategy was subsequently applied to more complex macromolecular architectures, including PCL_r_-*b*-PGMA_n_ block copolymers and statistical amphiphilic copolymers, where glycidyl units were integrated within a P­(PEGMA) block, resulting in structures of the type of PCL_r_-*b*-[P­(PEGMA)_m_-*co*-PGMA_n_]. PCL_r_-*b*-PGMA_n_ block copolymers were prepared using the same polymerization technique and reaction conditions adopted for PGMA homopolymer synthesis, but with an in-house-produced PCL_r_-Br macroinitiator instead of the commercial EBiB.^48^ PCL_r_-Br was obtained via bulk ring-opening polymerization (ROP) of ε-caprolactone (monomer conversion χ_ROP_ ≥ 98%, calculated via ^1^H NMR analysis). The terminal hydroxyl groups of the PCL chains were quantitatively converted into isobutyryl bromide end groups to generate ATRP macroinitiators for subsequent polymerization steps (detailed synthesis procedure reported in the Supporting Information). In a similar manner, PCL_r_-*b*-[P­(PEGMA)_m_-*co*-PGMA_n_] macromolecules were obtained via random copolymerization of varying ratios of commercially available PEGMA (average *M*_n_ = 500 Da) and GMA, using Cu­(I)­Br/HMTETA as the catalytic system and PCL_r_-Br as the macroinitiator.^53^ The detailed synthetic pathway of the various copolymers is reported in Figures S2 and S3. Both PGMA and P­(PEGMA)-*co*-PGMA blocks achieved the target DP and molecular weight, with ATRP reaching high conversions (χ_ATRP_ ≥ 96% after 18 h) for both monomers, as confirmed by ^1^H NMR. The DP of PGMA_n_ was set equal to 30 units for both homopolymers and PCL_r_-*b*-PGMA_n_ diblock copolymers. In PCL_r_-*b*-[P­(PEGMA)_m_-*co*-PGMA_n_] polymers, the number of GMA units was tuned to not exceed 20% of the total length of the P­(PEGMA)-*co*-PGMA segment. Likewise, the hydrophobic PCL_r_ block was synthesized with a constant 30 caprolactone units across all copolymers. The lengths of the PCL block and the PGMA or P­(PEGMA)-PGMA segments were selected in order to obtain a sufficiently flexible functionalized copolymer that could form micellar NPs stable in aqueous suspension, with a core large enough to encapsulate hydrophobic drugs.^48,53,54^ The molecular structures of the synthesized copolymers are highlighted in Figure 2a.

**Figure 2:**
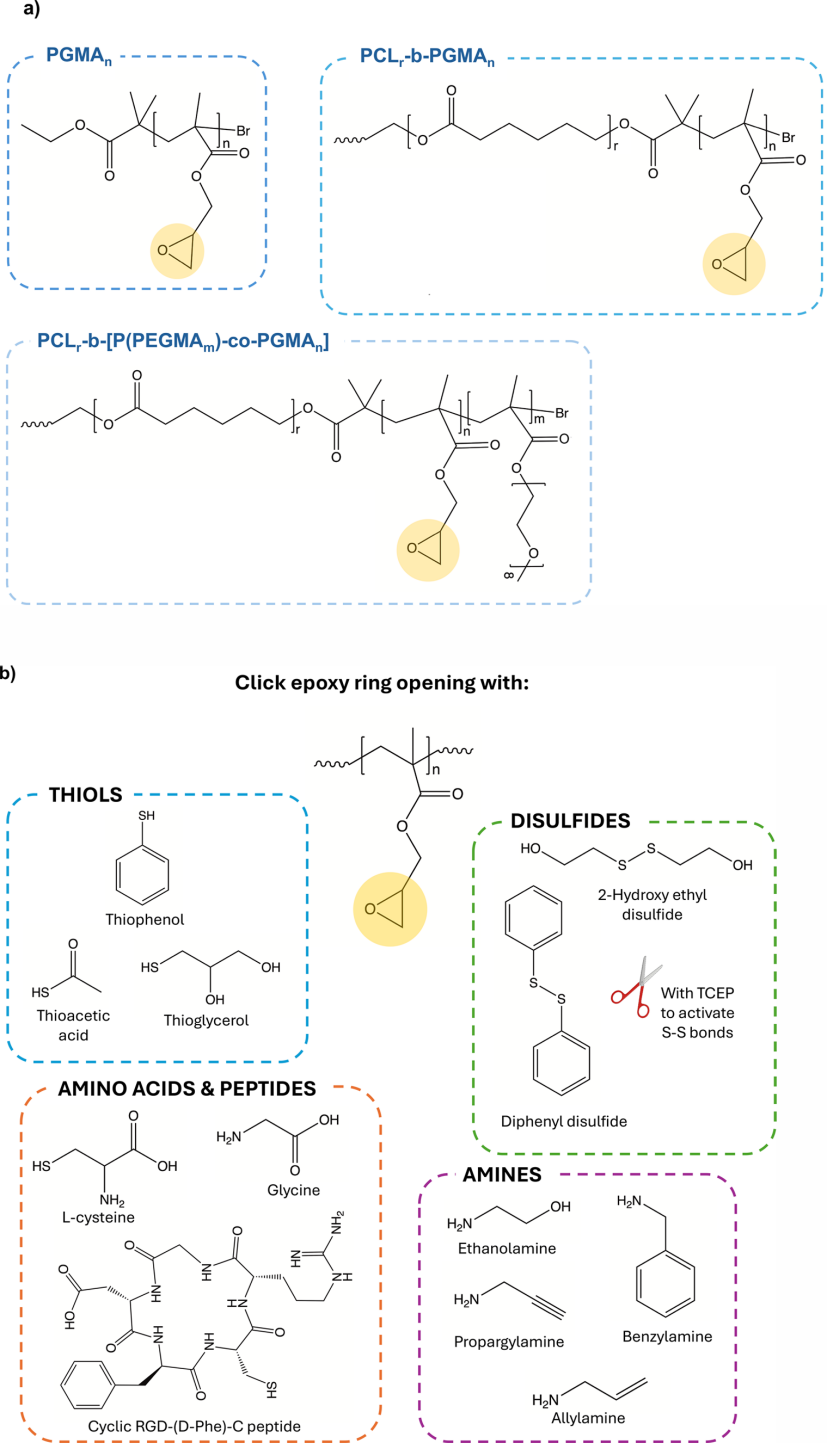
(a) Macromolecular structures of the PGMA-containing polymers tested for postpolymerization modification with thiols, disulfides, and amines. (b) List and classification of the reactive molecules and their molecular structures tested as modifying agents for the glycidyl ring-opening reaction.

The list of the studied macromolecules with their characterization data is summarized in Table 1, and the ^1^H NMR spectra of the purified polymers are reported in Figure S4, together with their SEC chromatograms Figure S5. The discrepancy between the *M*_n_ determined via ^1^H NMR and that obtained with SEC can be attributed to the limitations of polystyrene-based calibration standards, as well as to the inherent constraints of SEC in accurately assessing the molecular weight of comb-like polymer architectures, as previously reported in the literature.^34,48,53^ Both PGMA_n_ homopolymers and copolymers endowed with PGMA_n_ blocks were treated with molecules containing free −SH groups or S–S bonds. Similar parameters were then used to test analogue reactions involving amine-bearing molecules. The list of all molecules tested as functionalizing agents is provided in Figure 2b, along with their molecular structures.

**Table 1:** Summary of Properties of PGMA_n_ Homopolymers, PCL_r_-*b*-PGMA_n_ and PCL_n_-*b*-[P­(PEGMA)_m_-*co*-PGMA_n_] Copolymers[Fn fn1]

Polymers	n	r	m	*M*_n,NMR_ [kDa]	*M*_n,SEC_ [kDa]	Đ [-]
PGMA_n_	30	-	-	4.4	5.7	1.1
PCL_r_-*b*-PGMA_n_	30	30	-	7.7	9.2	1.2
PCL_r_-*b*-[P(PEGMA)_m_-*co*-PGMA_n_]	3–6	30	29	18.5	20.7	1.2

### 3.2 PGMA_n_ Homopolymers Postpolymerization Modification with Thiolated Molecules

Due to the interest in potential biological applications, thioacetic acid and thioglycerol were selected to initially test the GMA functionalization potential. Thioacetic acid demonstrated to be particularly useful as its reaction with the glycidyl scaffolds introduces −SCOOCH_3_ moieties into the polymer. These end-chain functionalities can subsequently undergo mild basic hydrolysis to yield free −SH groups within the macromolecule, allowing for their selective activation when required.^48^ Indeed, thiolated macromolecules and nanocarriers have been proposed to enhance the mucoadhesive properties of various drug delivery systems, owing to their capacity to establish covalent interactions with cysteine-rich domains of mucin glycoproteins.^33,48,55,56^ Under physiological conditions, these materials can undergo disulfide-thiol exchange reactions, closely resembling native disulfide linkages within the mucosal barrier. These covalent interactions are significantly stronger than the noncovalent forces typically observed with nonthiolated polymers.^57–59^ On the other hand, functionalization with thioglycerol led to the synthesis of poly­(2-hydroxy-3-(thioglycerol)­propyl methacrylate) (PTGMA), particularly beneficial for achieving hydrophilicity from native PGMA. In this light, the strategy enables the synthesis of block copolymers via ATRP in organic solvents, where a hydrophobic PGMA block can be successively modified to enhance its polarity, making it soluble in aqueous solvents. This approach is useful for creating amphiphilic macromolecules with a hydrophobic block (e.g., PCL) and a hydrophilic one, which can then self-assemble into core–shell structures, largely employed as drug delivery tools in various biomedical applications. Therefore, using a hydrophilic shell made of PTGMA could serve as a viable alternative to the more commonly used PEGMA, with the advantage of presenting a lower molar mass and reduced steric hindrance of the monomeric unit. At the same time, this method offers an alternative to the ATRP of glycerol monomethacrylate, which is not particularly suitable for the synthesis in common organic solvents (as THF) due to its polarity, especially when polymerized onto hydrophobic macroinitiators. The postpolymerization modification of PGMA_n_ with thioacetic acid was successfully carried out both in THF and DMF, achieving 100% conversion with a molar ratio of epoxy rings/thioacetic acid = 1/1. Similarly, quantitative conversion was achieved in the functionalization of PGMA with thioglycerol in DMF. DMF was selected as one of the solvents tested due to its excellent ability to solubilize both the relatively nonpolar PGMA backbone and more polar molecules such as thioglycerol, as well as biologically active compounds, including peptides and amino acids. Its amphiphilic nature and high dielectric constant allow the efficient dissolution of diverse chemical species without compromising the structural integrity or biological activity of sensitive functional groups. In contrast, aqueous buffer systems were not suitable due to the poor aqueous solubility of PGMA, which would hinder homogeneous reaction conditions and efficient functionalization. For both reactions presented the temperature was set at 25 °C and an excess of TEA was employed as catalyst (details of the reaction conditions are reported in Table 2). The ^1^H NMR spectra (in DMSO-*d*_6_) of both native PGMA and modified PGMA with thioacetate and thioglycerol are shown in Figure 3, along with the final structure of the resulting molecules. The reported^1^ H NMR spectra (designated as A and B, corresponding to thioacetic acid and thioglycerol functionalization, respectively) demonstrate the disappearance of the epoxide proton signals (2.5–3.0 ppm, labeled “C” in the native PGMA_n_ spectrum) following chemical modification. This disappearance is accompanied by the emergence of new resonances attributed to the functional groups. Specifically, in the case of thioacetic acid conjugation, a distinct signal at 2.0 ppm corresponds to the methyl protons of the −SCOCH_3_ moiety (labeled “E”), while a resonance at 5.3 ppm is assigned to the hydroxyl proton generated via epoxide ring opening. Similarly, for thioglycerol functionalization, the appearing peaks in the region of 4.4–5.4 ppm correspond to the newly generated −OH functionalities, originated from the epoxy ring opening and from thioglycerol itself; while the peaks labeled as ″G”, ″H”, and ″F” represent the protons of grafted thioglycerol. In parallel, thiophenol was selected as a model compound to systematically optimize the reaction conditions for complete modification. Its choice was motivated by the ability to efficiently monitor functionalization progress via ^1^H NMR, owing to the distinct visibility of the benzyl group in the left region of the spectrum. This approach confirmed that even without an excess of TEA (molar ratio epoxy ring/thiophenol/TEA = 1/1/1), the reaction proceeded to complete conversion. A list of all reaction conditions tested is provided in Table 2.

**Figure 3:**
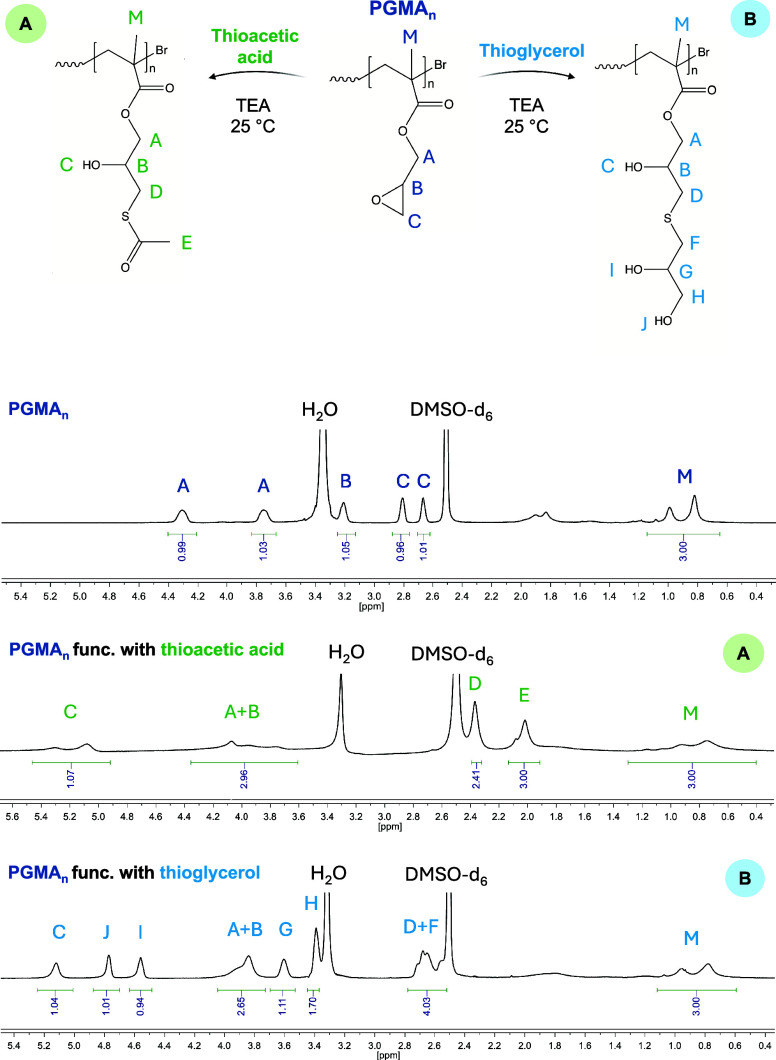
Comparison of ^1^H NMR spectra (in DMSO-*d*_6_) recorded: before PGMA_n_ modification; after the oxirane opening reaction carried out with thioacetic acid at 25 °C (epoxy ring/thioacetic acid/TEA = 1/1/3) (A); after the epoxy ring-opening reaction performed with thioglycerol at 25 °C (epoxy ring/thioglycerol/TEA = 1/1/3) (B).

**Table 2:** Summary of the Different Reaction Conditions Tested for GMA Modification via Epoxy Ring Opening Operated by Thiolated Molecules and Amines[Fn fn2]

Functionalizing agent	Solvent	Red. agent	*T* [°C]	Epoxy ring/Funct. ag./Catalyst	χ_Funct._ [%]
**Thioacetic acid**	THF	-	25	1/1/3	100
	DMF	-	25	1/1/3	99
**Thioglycerol**	DMF	-	25	1/1/3	100
	DMF	-	25	1/1/1	100
	DMF	-	25	1/0.9/3 (cofunct. with thiophenol)[Fn fn3]	100
	DMF	-	25	1/0.9/3 (cofunct. with cRGD)[Fn fn3]	100
**Thiophenol**	THF	-	25	1/1/3	100
	THF	-	25	1/1/2	100
	THF	-	25	1/1/1	99
	DMF	-	25	1/1/1	99
	THF	-	25	1/0.1/3 (cofunct. with thioglycerol)[Fn fn3]	100
**Propargylamine**	DMF	-	25	1/1/3	0
**Allylamine**	DMF	-	25	1/2/3	<3
	DMF	-	25	1/1/3	0
**Glycine**	DMF	-	25	1/2/3	0
	DMF	-	25	1/1/3	0
**Benzylamine**	TEA	-	25	1/2/3	18 ± 2
	TEA	-	60	1/2/3	27 ± 3
	TEA	-	25	1/1/3	4 ± 2
	TEA	-	60	1/1/3	14 ± 2
	TEA	-	25	1/1/1	0
	TEA	-	60	1/1/1	3 ± 2

Multiple functionalization reactions were also tested, where, at each step, a specific percentage of glycidyl rings was selectively functionalized with a chosen functionalizing agent. The goal was to extend this conjugation strategy to complex amphiphilic molecules, in order to develop multifunctional (micellar) nanocarriers capable of ensuring predictable *in vitro* and *in vivo* behavior, providing the desired interaction with biological systems. To this end, an initial proof-of-mechanism study was conducted by first selectively functionalizing only 10% of a PGMA polymer with thiophenol, followed by complete functionalization of the remaining native epoxy rings with thioglycerol. Both reaction steps resulted in quantitative functionalization, while maintaining the reaction system at 25 °C for 18 h. The ^1^H NMR spectra collected to monitor the progression of GMA unit modification are shown in Figure S6, along with the molecular structures obtained at different reaction stages.

### 3.3 PGMA Postpolymerization Functionalization with Amine-Bearing Molecules

The findings highlighted in Section 3.2 pave the way for polymer functionalization by means of biologically relevant molecules containing active thiols, such as amino acids or peptides. However, these molecules often contain amine groups within their structures, which can compete with thiols in binding to glycidyl rings. Despite that epoxy ring opening by amines generally requires longer reaction times and higher temperatures,^3,6,7,32,44^ various amine-containing molecules were tested for the modification of PGMA chains, assessing how different reaction conditions (temperature, molar ratios), amine molecular weight, solubility, and functional groups could influence their reactivity. As shown in Table 2, the functionalization of PGMA with propargylamine under the conditions typically employed for thiol functionalization resulted in no detectable conversion (^1^H NMR reported in Figure S7a). Similarly, when glycine (an amino acid containing only an amine group) was used as a functionalizing agent, no improvements in terms of conversion were observed, even when doubling the amount of glycine employed for the reaction and maintaining an excess of catalyst (^1^H NMR shown in Figure S7b).

More extensive studies were conducted using benzylamine, given its structural similarity to thiophenol, which was used as a reference compound in the previous section. Several experiments were performed, systematically increasing the temperature to 60 °C and progressively increasing the molar amounts of both the catalyst and the functionalizing agent. The results, summarized in Table 2, demonstrate that elevated temperature, along with increased amounts of catalyst and benzylamine, positively influenced the conversion. However, under the conditions designed for thiol-based functionalization, the conversion achieved with benzylamine remains negligible (compared to the complete modification observed with sulfur-containing compounds).

The ^1^H NMR spectrum of the PGMA homopolymer functionalized with thiophenol is shown in Figure S8, where it is compared to the spectrum achieved after PGMA functionalization with benzylamine. With complete PGMA functionalization with thiophenol, the peak corresponding to the benzyl group clearly appears in the region of 7.0–7.5 ppm, together with the typical peak of the −OH group (5.3 ppm) arising from epoxide ring opening (Supporting Information).

### 3.4 PGMA Postpolymerization Functionalization with Disulfide-Bearing Molecules

The same functionalization mechanism studied in the presence of free thiols was tested using R-S-S-R type molecules, which contain a disulfide bridge linking two identical units (R). In this case, the approach involved initially treating the disulfide-containing molecule with a reducing agent to generate two nucleophilic R-S^–^ subunits, which are then reactive and capable of modifying the epoxide rings of PGMA, following a pathway similar to the one presented in Section 3.2. In light of this, R-S-S-R molecules were treated with TCEP as a reducing agent. In this study, it was investigated whether TCEP alone could not open the epoxide rings within the mild reaction conditions applied, treating PGMA with both equimolar and excess amounts of TCEP. ^1^H NMR spectrum shown in Figure S9 confirmed that the PGMA_n_ structure remained unaltered after treatment with TCEP and TEA, both in DMF and THF (epoxy ring/TCEP/TEA = 1/1/3). Thus, TCEP was revealed to be a suitable reducing agent to functionalize PGMA with disulfide-bearing molecules without altering the polymer’s molecular integrity or reaction outcome.

Therefore, the PGMA homopolymer was modified with 2-hydroxyethanethiol by exploiting 2-hydroxyethyl disulfide, following the cleavage of its S–S bond mediated by TCEP.

Initially, 2-hydroxyethyl disulfide and TCEP were separately dissolved in DMF and then mixed to generate free OHCH_2_CH_2_–SH groups (2-hydroxyethyl disulfide/TCEP = 1/0.5). The resulting mixture was subsequently reacted with PGMA in the presence of TEA in DMF. The reaction proceeded under mild conditions at 25 °C overnight, leading to the complete functionalization of the glycidyl scaffolds (Table 3). The reaction pathway followed by the ^1^H NMR spectrum of the resulting polymer and the corresponding chemical structure is reported in Figure S10. Despite the additional sulfhydryl activation step, the modified molecule obtained closely resembles the ones previously achieved with thiolated molecules, especially when thioglycerol was selected. PGMA original peaks, labeled as “A”, “B”, and “C” (Figure 3, PGMA_n_ homopolymer spectrum), disappear in favor of new groups: in the region of 4.5–5.5 ppm, the −OH functionalities belonging to 2-hydroxyethyl disulfide and formed after epoxide rupture are clearly visible, while going from 2.5 to 3.7 ppm, peaks “D”, “E”, and “F” represent the three CH_2_ groups introduced with the attachment of OHCH_2_CH_2_–SH moiety to the previously existing GMA chain.

**Table 3:** Summary of the Different Reaction Conditions Tested for GMA Modification via Epoxy Ring Opening Operated by Disulfide-Bearing Compounds[Fn fn2]

Functionalizing agent	Solvent	Red. agent	*T* [°C]	Epoxy ring/Funct. ag./Catalyst	χ_Funct._ [%]
**2-Hydroxy ethyl disulfide**	DMF	TCEP	25	1/1/3	100
**Diphenyl disulfide**	THF	TCEP	25	1/1/3	100
	THF	TCEP	25	1/1/2	99
	THF	TCEP	25	1/1/1	99
	DMF	TCEP	25	1/1/1	99
	THF	-	25	1/1/3	0
	DMF	-	25	1/1/3	0
l **-cysteine**	DMF	TCEP	25	1/1/1	99
**cRGD peptide**	DMF	TCEP	25	1/0.1/3 (cofunct. with thioglycerol)[Fn fn3]	100

An aromatic-containing molecule (diphenyl disulfide) was selected as a model to test different reaction conditions and compare the resulting outcomes to those obtained by functionalizing PGMA with thiophenol. To cleave the disulfide link, diphenyl disulfide was dissolved in DMF along with TCEP, and the resulting mixture was used to treat PGMA. Maintaining the system at 25 °C enabled complete (100%) conversion, both when using an excess of TEA catalyst and when applying it in a stoichiometric ratio relative to the number of glycidyl rings and active functional groups (Table 3). The NMR spectrum obtained at the end of the reaction (Figure 4) was identical to that observed when directly reacting active thiophenol with PGMA under the same conditions (Section 3.2). The reaction was carried out in both THF and DMF, yielding comparable results. This observation is further supported by functionalization kinetics monitored via ^1^H NMR (Figure 4), which demonstrate that the choice of solvent does not significantly affect the reaction rate or overall efficiency (the ^1^H NMR recorded in the two solvents at different time points are reported in Figure S11). Finally, control experiments were conducted to verify that PGMA modification with diphenyl disulfide did not occur in the absence of TCEP both in THF and in DMF (Figure 4, bottom), as the lack of a reducing agent prevents the formation of free reactive thiols. As shown in the spectrum in Figure S12, the structure of PGMA remained completely unaltered, despite being allowed to react for 18 h at 25 °C in the presence of TEA and diphenyl disulfide in THF or DMF.

**Figure 4:**
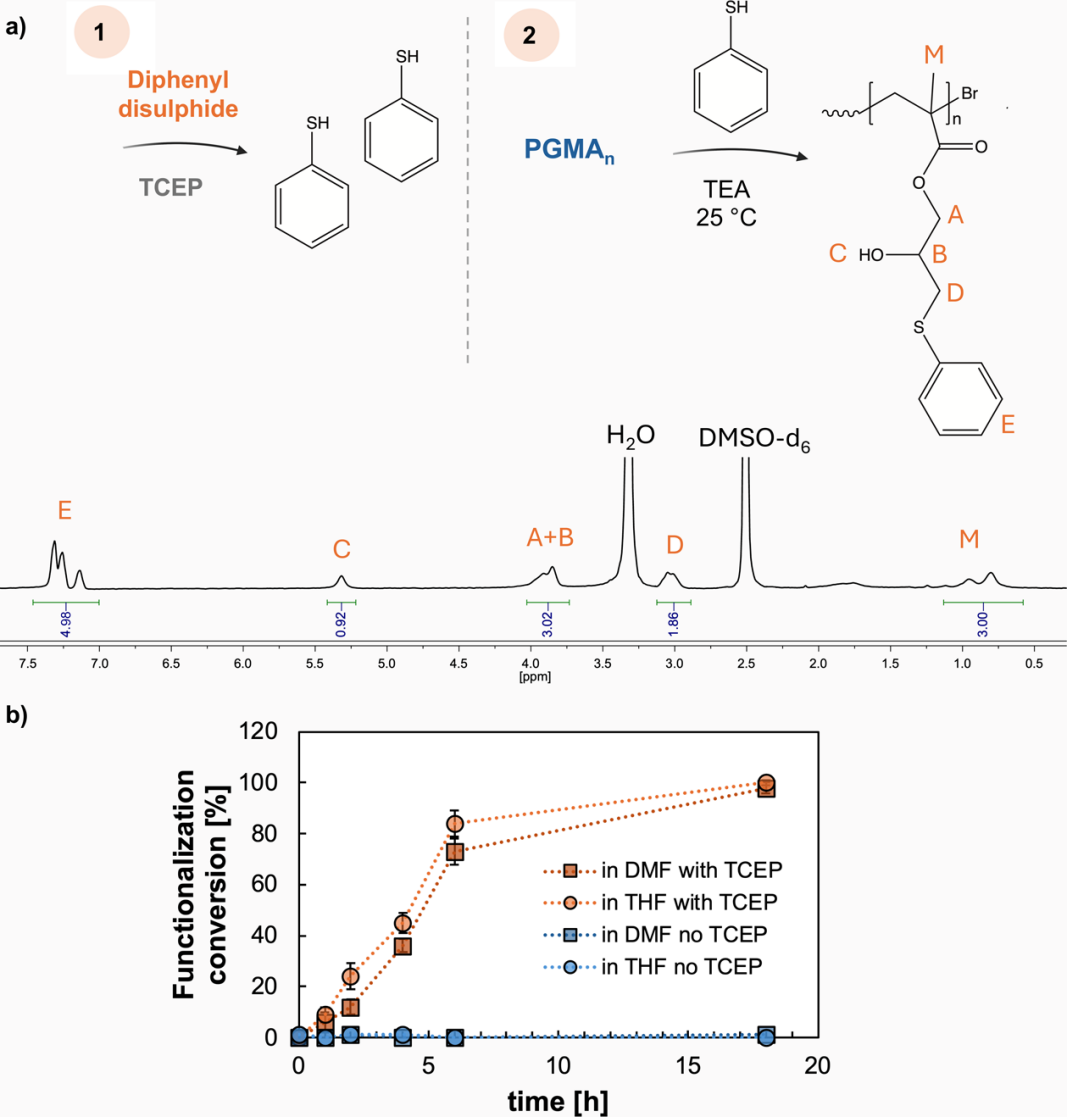
**a)** ^1^H NMR spectra (in DMSO-d_6_) of PGMA_n_ treated with diphenyl disulfide in the presence of TEA at 25 °C (epoxy ring/TEA = 1/1), when reduced by TCEP. **b)**Kinetic profile of the functionalization reaction occurring in DMF (squares) and THF (circles), in the presence of TCEP (orange) or not (blue), highlighting the inability to functionalize PGMA with diphenyl disulfide without a reducing agent.

### 3.5 Optimized Reaction Conditions and Chemoselectivity Study

Additional confirmation of the more straightforward functionalization in the presence of thiols, compared to amines, was obtained using l-cysteine as a modifying agent. Prior to the reaction, the amino acid (containing both −SH and −NH_2_ groups) was treated with TCEP to reduce any disulfide bonds that may have formed during storage or handling of the compound. It was then reacted with PGMA and TEA for 18 h at 25 °C (Table 3). Analysis of the resulting product via ^1^H NMR (provided in both DMSO-*d*_6_ and D_2_O, Figure 5) revealed that all detected peaks corresponded in both chemical shift and integration area to those expected for thiol-selective conjugation, with no evidence of amine-mediated attachment. If the amine had participated in the reaction, distinct peaks would have appeared due to changes in the chemical environment of equivalent protons. To further confirm the absence of free thiol groups in the functionalized polymer (potentially arising from cysteine attachment via the amine, rather than the thiol), the sample was treated with an immobilized TCEP disulfide-reducing gel and subsequently analyzed using Ellman’s reagent (DTNB) assay^60^ to quantify free -SH groups. UV–vis analysis performed at 412 nm showed negligible absorbance, indicating the complete absence of free sulfhydryl groups in the modified polymer (Figure S13).

**Figure 5:**
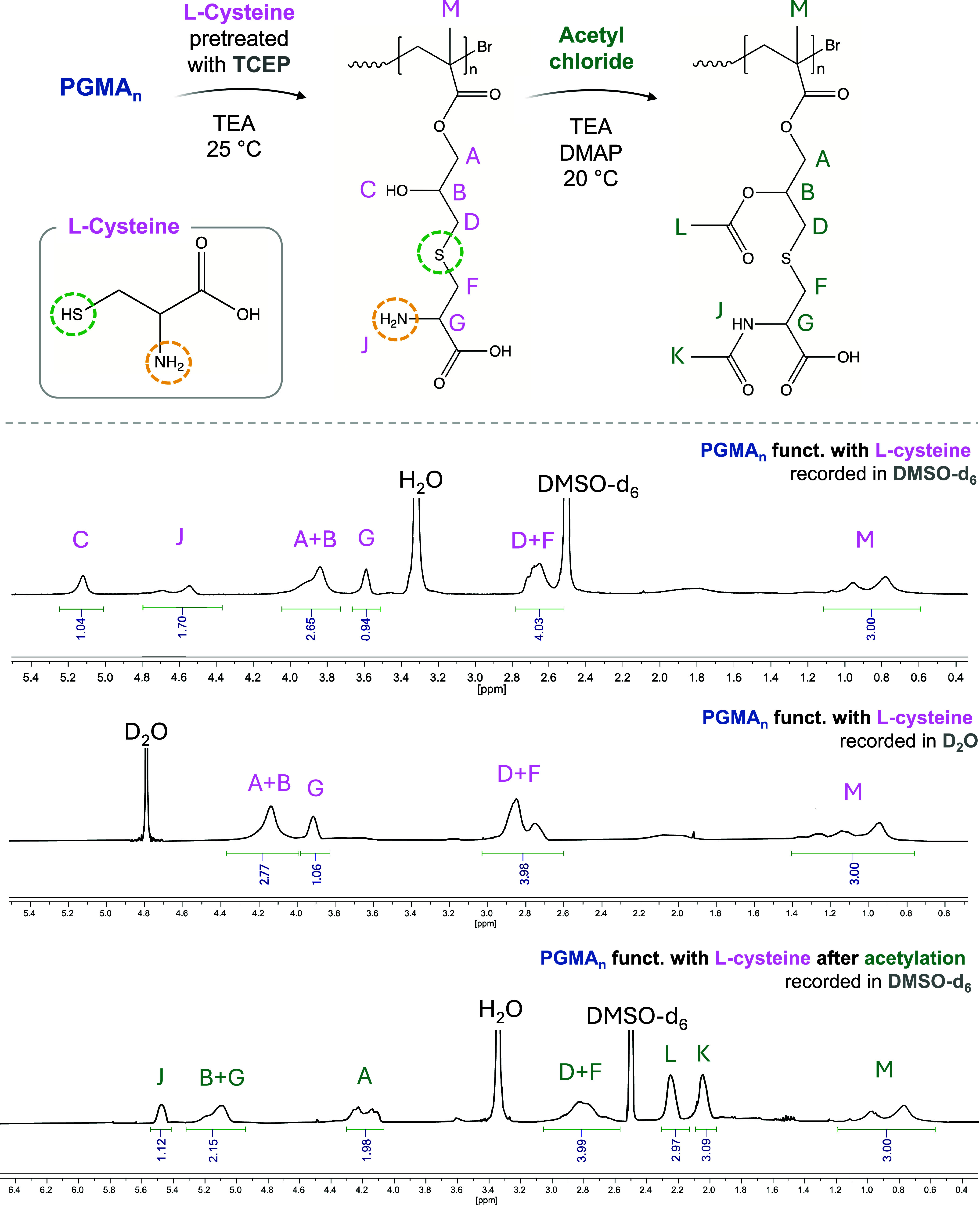
^1^H NMR spectra (in DMSO-*d*_6_ and in D_2_O) of PGMA_n_ reacted with l-cysteine in the presence of TEA at 25 °C (epoxy ring/l-cysteine/TEA = 1/1/1) for 18 h, formerly treating l-cysteine with TCEP to reduce possible S–S bonds present in the system. ^1^H NMR spectra (in DMSO-*d*_6_) of cysteine-functionalized PGMA_n_ were acetylated with an excess of acetyl chloride to induce the formation of an amide and an ester group.

Additionally, cysteine-modified PGMA was reacted with acetyl chloride in the presence of TEA and DMAP to induce acetylation of both the hydroxyl group resulting from the epoxide ring opening and the primary amine introduced via cysteine conjugation. This reaction led to the formation of the acetyl ester and acetamide functionalities. The signals corresponding to these newly introduced groups appear as very sharp peaks at distinct chemical shifts (2.0 and 2.3 ppm, denoted as “L” and “K”, respectively). Their integration (area = 3, corresponding to the 3H^+^ of the methyl groups) clearly quantifies the degree and orthogonality of the functionalization. Accordingly, the signals assigned to the carbon atoms labeled “B” and “G” prior to acetylation appear shifted to the 5.0–5.3 ppm region. The signal previously attributed to the free hydroxyl group formed upon epoxide ring opening (“C”) is no longer detectable, indicating successful acetylation. Furthermore, the primary amine (“J”) introduced by cysteine is now observed as an NH group, showing both a chemical shift and a change in integration, consistent with its conversion to an acetamide.

In contrast, the signals corresponding to the methylene groups adjacent to the sulfur atom (“D”, “F”) in the modified PGMA backbone remain unchanged, confirming the site-specific nature of the acetylation and the chemical stability of the sulfur-containing moiety.^40,42,61^

Table 4 provides an overview of the variation in the molecular weight of native PGMA_30_ compared to its derivatives functionalized with different thiolated molecules, further confirming successful functionalization. A clear increase in *M*_n_ is observed, as highlighted in Figure 6, which displays the chromatograms of both native PGMA and its counterparts modified with thioglycerol and l-cysteine as representative examples. Additionally, the dispersity of the analyzed polymers remains relatively low, with only a slight variation compared to the starting polymer.

**Table 4:** *M*_n,NMR_ and *M*_n,SEC_ of Native PGMA_30_ and of Its Modified Version with Thiolated Compounds

Polymers	*M*_n,NMR_ [kDa]	*M*_n,SEC_ [kDa]	Đ [-]
PGMA_30_	4.4	5.7	1.1
PGMA_30_ mod. with thioacetic acid	6.7	7.4	1.3
PGMA_30_ mod. with thiophenol	7.8	10.2	1.3
PGMA_30_ mod. with thioglycerol	7.7	11.3	1.4
PGMA_30_ mod. with l-cysteine	7.1	8.2	1.3

**Figure 6:**
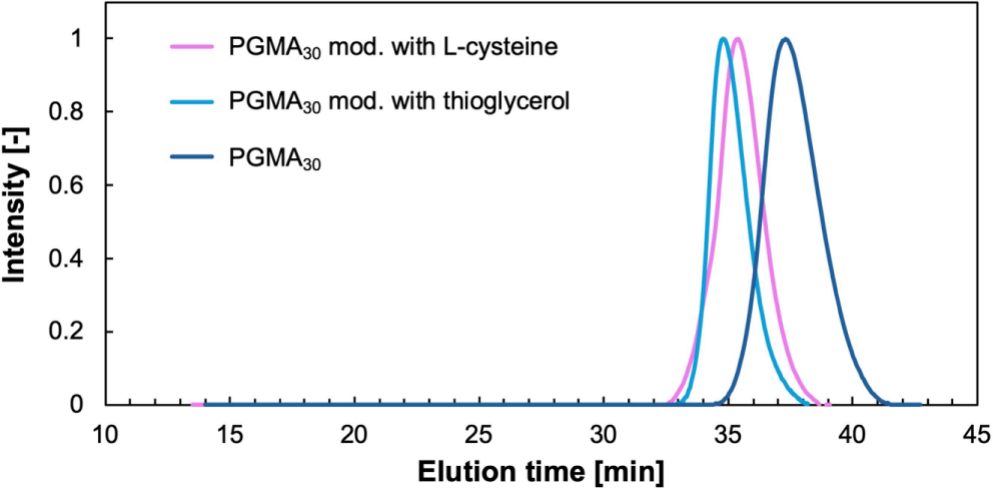
SEC chromatograms of native PGMA_30_ and of the modified polymer with thioglycerol and l-cysteine.

### 3.6 Selective Conjugation of Glycidyl-Bearing Copolymers

After a systematic investigation of the reactivity of thiols and amines with the epoxy groups of PGMA homopolymers, the focus was extended to more complex molecular architectures, such as amphiphilic block copolymers for biomedical use.

Previous studies have demonstrated that GMA units statistically copolymerized with PEGMA, forming PCL_r_-*b*-[P­(PEGMA)_m_-*co*-PGMA_n_] copolymers (Figure 2a), can be successfully functionalized with thiols (*n* < 20% × (*m* + *n*)), in particular with thioacetic acid.^48^ The thioester moieties formed after PGMA functionalization were deprotected by removing the acetyl group through hydrolysis, yielding thiolated polymers with both tailored mucoadhesive properties and the ability to form disulfide bonds within the NP corona, conferring strength and enhanced stability to the NPs. These copolymers also achieved complete functionalization using thiophenol as a reference molecule, using the reaction conditions described in the previous sections (^1^H NMR spectra and the chemical structure of the resulting macromolecule are shown in Figure 7).

**Figure 7:**
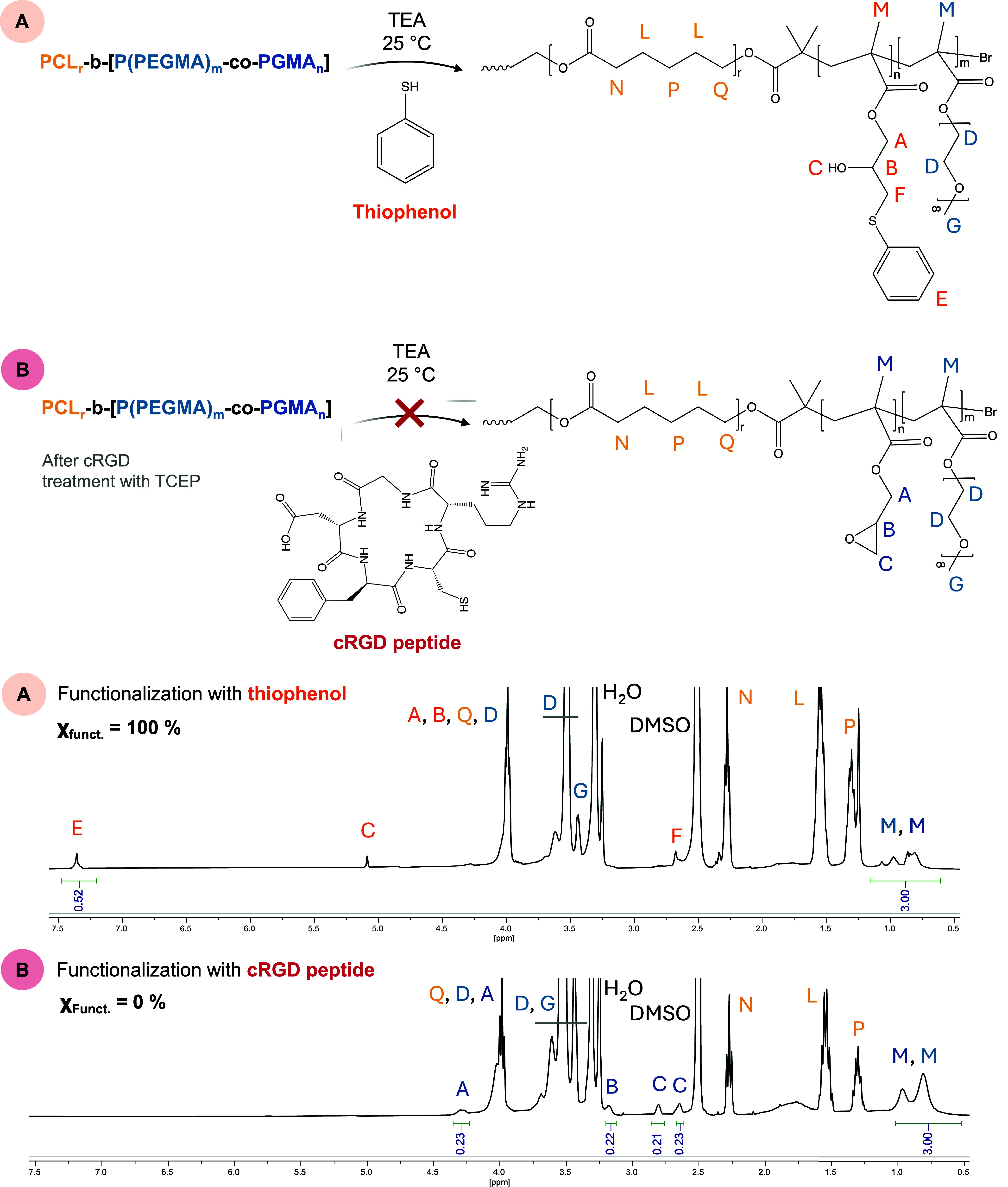
^1^H NMR spectra (in DMSO-*d*_6_) of PCL_r_-*b*-[P­(PEGMA)_m_-*co*-PGMA_n_] reacted with (A) thiophenol in the presence of TEA at 25 °C (epoxy ring/thiophenol/TEA = 1/1/3) for 18 h (PGMA_n_ represents 10% of the P­(PEGMA)_m_-*co*-PGMA_n_ block (*n* = 3, *m* = 29)); (B) cRGD peptide in the presence of TEA at 25 °C (epoxy ring/cRGD/TEA = 1/1/3) for 18 h (PGMA_n_ represents 20% of the P­(PEGMA)_m_-*co*-PGMA_n_ block (*n* = 6, *m* = 29)).

In contrast, the modification of glycidyl units of these copolymers with higher molecular weight molecules such as peptides proved particularly challenging. This difficulty arises from the steric hindrance imposed by the PEGMA segments, which may obstruct the access of these functionalizing agents to the epoxy ring. Several attempts were performed to functionalize PCL_r_-*b*-[P­(PEGMA)_m_-*co*-PGMA_n_] copolymers with a modified cyclic form of the RGD peptide (cRGD) presenting Arg-Gly-Asp-(d-Phe)-Cys) amino acid sequence. cRGD is commonly used as a model peptide due to its relevance in nanomedicine as a targeting ligand for tumor cells, angiogenic endothelial cells, the placenta, and the kidney glomerulus.^35,62,63^ Even upon increasing the number of GMA units (from *n* = 10% × (*n* + *m*) to *n* = 20% × (*n* + *m*)), to assess whether a higher density of reactive sites within the PEGMA block would facilitate peptide conjugation, no functionalization occurred. In all cases, the ^1^H NMR spectra of the treated polymers remained unchanged, with no detectable variation in the characteristic peaks of the epoxy ring within the copolymer structure and with no signal attributable to the presence of the peptide (Figure 7). The latter reaction was carried out in DMF, despite the presence of PEGMA units, which could, in principle, provide solubility in aqueous buffer systems. This choice was made because glycidyl groups were found to be unstable in the presence of water, undergoing spontaneous ring-opening even in the absence of conjugation with the target molecule. Therefore, this side reaction may compromise the integrity of the reactive epoxy moieties. Comparative ^1^H NMR spectra of the PCL_r_-*b*-[P­(PEGMA)_m_-*co*-PGMA_n_] copolymer before and after dialysis against water support these findings and are presented in Figure S14.

The study was extended to the use of PTGMA as the hydrophilic block of the copolymers, which could serve as a viable alternative to the commonly used P­(PEGMA), with the advantage of presenting a lower molar mass and reduced steric hindrance of the monomeric unit. Starting from a PCL_r_-*b*-PGMA_n_ copolymer (with *r* = *n* = 30) (Figure 2a), the entire PGMA block was functionalized with thioglycerol to obtain PCL_r_-*b*-PTGMA_n_, i.e., an amphiphilic copolymer featuring a hydrophobic PCL block and a hydrophilic thioglycerol-modified PGMA segment. The conjugation reaction was performed under the same conditions presented in Section 3.2, but with the amount of TEA reduced to equimolar quantities relative to the epoxy rings, based on the optimization carried out in previous tests (Table 2).

PCL_30_-*b*-PTGMA_30_ was then used to formulate NP suspensions in aqueous buffer according to a nanoprecipitation/solvent evaporation method.^34,48^ The micelles were suspended in PBS at pH 7.4 and were characterized via DLS analysis. The ^1^H NMR spectra of the final polymer, the Z-average size, and the polydispersity index of the corresponding NPs are reported in Figure S15 together with the size distribution curve. The NPs presented a monomodal size distribution (average particle size: 43 nm), exhibiting a single dominant peak accounting for 100% of the scattering intensity and a PdI < 0.2, confirming narrow size distributions and high homogeneity.

In order to achieve conjugation of PCL-*b*-PTGMA copolymers with bioactive moieties, the cofunctionalization strategy applied to PGMA_n_ homopolymers (Section 3.2) was extended to PCL_r_-*b*-PGMA_n_ copolymers. In this case, 10% of the PGMA block was functionalized with the cRGD peptide, previously tested on PCL_r_-*b*-[P­(PEGMA)_m_-*co*-PGMA_n_], while the remaining portion was modified with thioglycerol. The two reactions were carried out sequentially without intermediate purification. First, the cRGD peptide was treated with TCEP dissolving both molecules in DMF, and then, PCL_r_-*b*-PGMA_n_ was reacted with the cRGD-TCEP solution and TEA for 18 h at 25 °C (Table 3). Upon completion of this reaction, the required amount of thioglycerol was added to ensure the complete opening of the remaining epoxy rings. This approach endowed the macromolecule with biological activity through the incorporation of the cRGD peptide, while its amphiphilic nature enabled self-assembly into NPs, making it suitable for drug delivery applications. Since the cRGD peptide targets cancer cells and tumor vasculature via integrin binding, cRGD-functionalized NPs may promote selective binding and precise drug release for improved therapeutic performance.^64^ In particular, peptide cyclization preorganizes the molecule into a conformation that more closely resembles the natural ligand, thereby enhancing its binding affinity compared to the linear RGD sequence and improving selectivity toward specific integrin subtypes. The cyclic structure also confers significantly greater stability against enzymatic degradation, prolonging the peptide’s half-life in circulation. At the same time, reduced conformational flexibility improves receptor-binding availability and tissue penetration. Finally, the incorporation of a d-amino acid (d-phenylalanine) further increases the proteolytic stability and contributes to the optimal cyclic conformation.

The ^1^H NMR analysis (Figure 8) of the resulting copolymer reveals a spectrum closely resembling the one previously obtained for PCL_r_-*b*-PGMA_n_ functionalized solely with thioglycerol (Figure S15). However, an additional peak appears in the region above 7 ppm (characteristic of aromatic signals), with an integration area matching the expected contribution of the phenylalanine present in the peptide (labeled as “E”). Simultaneously, increased integration areas are observed for the peaks in the regions 2.6–3, 3.5–4.0, and 4.5–5.5 ppm, which can be attributed to the multiple −CH, CH_2_, −OH, and −NH groups belonging to the cRGD peptide. The functionalized macromolecules were employed to formulate micellar NPs according to the method discussed above. The ^1^H NMR spectra of the resulting polymer, the Z-average size, and the polydispersity index of the corresponding NPs are reported in Figure 8, together with the size distribution curve. The obtained NPs exhibit a slightly broader size distribution than those previously observed composed solely of PCL-*b*-PTGMA, likely due to the steric hindrance introduced by the peptide. Nonetheless, they maintain a unimodal distribution and an average size comparable to those of the earlier results.

**Figure 8:**
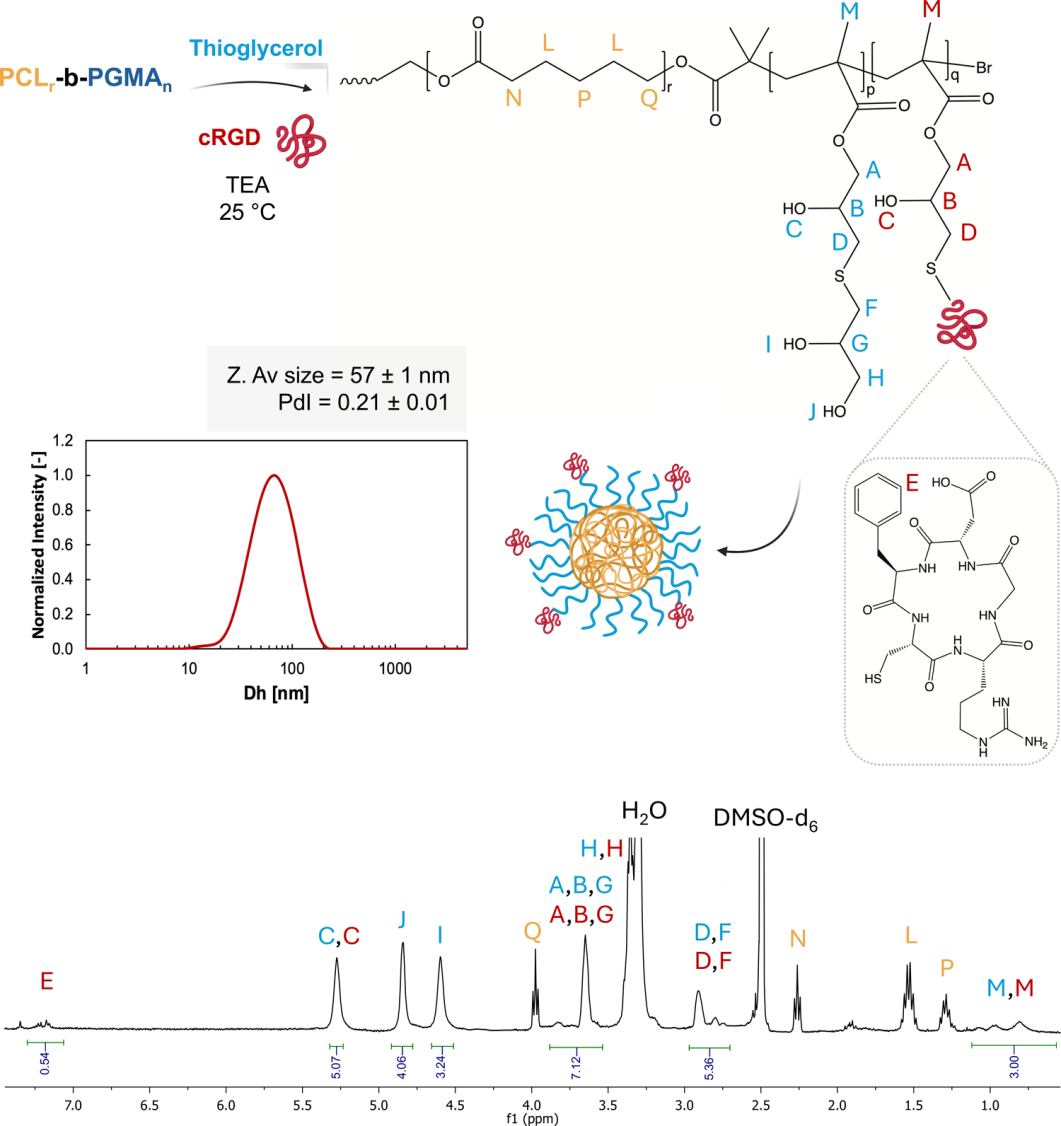
PCL_r_-*b*-PGMA_n_ modification with thioglycerol and cRGD peptide in two steps (18 h each, 25 °C) where TEA was inserted in excess at the first stage and not in the second one (epoxy ring/thioglycerol/cRGD/TEA = 1/0.9/0.1/3; *p* = 0.9·*n* and *q* = 0.1·*n*). Amphiphilic and biologically active molecules were obtained and able to produce core–shell micelles in aqueous suspension (10 mg/mL in PBS 10 mM, pH 7.4). Size distribution curve, Z-average size, and PdI were evaluated by DLS analysis (top). In the bottom, ^1^H NMR spectra (in DMSO-*d*_6_) of the resulting macromolecule.

The proposed method for obtaining multifunctional nanocarriers is particularly advantageous, as it enables two consecutive modifications using equimolar reactants (relative to the epoxy rings), minimizing product loss and optimizing the use of functionalizing agents, which is especially important for biologically active and costly compounds. Moreover, conjugating the molecule with the highest steric hindrance first simplifies the process and ensures a more efficient modification. Indeed, this approach is more advantageous than incorporating a large molecule, such as a peptide, into a copolymer with glycidyl units embedded in a preformed hydrophilic block, especially when the block consists of high-molecular-weight monomers such as PEGMA.

In summary, by adopting the proposed strategy based on the sequential functionalization of PCL_r_-*b*-PGMA_n_, the resulting nanocarriers successfully achieve both the intended biological functionality and amphiphilic character in a streamlined and efficient manner. The process is conducted under exceptionally mild reaction conditions that preserve the structural integrity of both the polymer backbone and the functional agent while ensuring quantitative conversion. Furthermore, the size of the resulting NPs is fully consistent with that of analogous systems synthesized by polymerizing hydrophilic monomers such as PEGMA or glycerol methacrylate from PCL-based macroinitiators with comparable design parameters.^35,48,65^

## 4 Conclusions

This study explored the selective functionalization of PGMA homopolymers and PCL-based block copolymers using thiol-epoxy chemistry under mild conditions. Using ATRP-synthesized PGMA_n_, various thiols, such as thioglycerol and thioacetic acid, were tested, achieving complete epoxide ring opening confirmed by ^1^H NMR. In particular, thioglycerol conjugation led to the synthesis of PTGMA, a highly hydroxylated polymer that imparts hydrophilicity and enables the formation of amphiphilic nanocarriers. Optimization with thiophenol showed quantitative conversion without a base excess. In contrast, functionalization attempts with amines (e.g., propargylamine, glycine, and benzylamine) yielded low to no conversion, even under harsher conditions, confirming the superior reactivity of thiols. Disulfide-containing molecules such as diphenyl and 2-hydroxyethyl disulfide were also employed using TCEP as a mild, selective reducing agent, achieving efficient thiol-based modification without compromising polymer structure. l-Cysteine was used to compare thiol and amine reactivity, revealing exclusive thiol conjugation and confirming the chemoselectivity of the reaction under the optimized working conditions. The strategy was extended to amphiphilic block copolymers (PCL-*b*-PGMA), producing NPs with narrow size distributions. Cofunctionalization was demonstrated by sequentially modifying PCL-*b*-PGMA copolymers with the cRGD peptide and thioglycerol, forming biologically active PTGMA-based nanosystems. The efficiency of this method, allowing sequential modifications with equimolar reactants, minimizes waste, which is especially advantageous when using costly biomolecules. The approach was also successfully applied to copolymers containing sterically hindered PEGMA segments. Overall, the findings highlight the potential of thiol-epoxy chemistry as a versatile and biocompatible alternative to thiol–ene reactions for postpolymerization modification. It eliminates the need for intermediate functionalization steps by using a reactive monomer, such as GMA as the main constituent. This also avoids the use of bulky hydrophilic monomers, which may hinder peptide functionalization as the required hydrophilicity can be provided by modified forms of PGMA itself. This provides a robust platform for developing multifunctional polymeric nanocarriers tailored for targeted drug delivery and biomedical applications.

## Supplementary Material



## Footnotes

i Number-average molecular weight obtained by ^1^H NMR (*M*_n,NMR_) and SEC (*M*_n,SEC_), respectively, with dispersity *Đ* (by SEC) are listed in the table. n, r, m, *M*_n_, and *Đ* are reported as mean values with a variability percentage below 5%.

ii All the reactions lasted for 18 h, always employing TEA as catalyst. χ_Funct._ represent the epoxy group functionalization conversion [%]. The reaction parameters and conditions reported are to be considered valid both for PGMA_n_ homopolymers and for copolymers containing a PGMA_n_ segment or a random block of P­(PEGMA)_m_ and PGMA_n_.

iii In the case of cofunctionalization where *X*% of a PGMA chain was selectively functionalized with the functionalizing agent *A* and the remaining *Y*% with agent *B*, the following molar ratios were applied: eq *A*/eq epoxy ring = *X*/100/1 for the first step, and eq *B*/eq epoxy ring = *Y*/100/1 for the second step. TEA was inserted in excess (epoxy ring/TEA = 1/3) during the first step, avoiding further addition during the second step. Specifically, thiophenol (10% of PGMA functionalization) was coupled with thioglycerol (90% of PGMA functionalization) in PGMA_n_, while cRGD peptide (10% of PGMA functionalization) was coupled with thioglycerol (90% of PGMA functionalization) in PCLr-*b*-PGMA_n_ (see Table 3).

## Notes

Synthesis and purification procedure of: PCL_r_, PGMA_n_ homopolymer, PCL_r_-*b*-PGMA_n_ block copolymer, and PCL_r_-*b*-[P­(PEGMA)_m_-*co*-PGMA_n_] copolymer; oxirane ring-opening pathway; reaction pathway for PGMA_n_, PCL_r_-*b*-PGMA_n_, and PCL_r_-*b*-[P­(PEGMA)_m_-*co*-PGMA_n_] synthesis via ROP and ATRP; ^1^H NMR spectra and SEC chromatograms of native PGMA_n_, PCL_r_-*b*-PGMA_n_, and PCL_r_-*b*-[P­(PEGMA)_m_-*co*-PGMA_n_] polymers; ^1^H NMR spectra recorded after treating PGMA_n_ with thioglycerol and thiophenol, benzylamine, propargyl amine, glycine, TCEP, 2-hydroxy ethyl disulfide (with TCEP), and diphenyl disulfide (with and without TCEP in THF and DMF); Ellman reagent assay output performed on PGMA_n_ modified with l-cysteine; ^1^H NMR spectra of PCL_r_-*b*-[P­(PEGMA)_m_-*co*-PGMA_n_] in its native form and after dialysis against water; ^1^H NMR spectrum, size distribution curve, Z-average size, and PdI by DLS of PCL_r_-*b*-PGMA_n_ modified with thioglycerol (PDF)
