# Supplementary material for: TCEP-Enabled Click Modification of Glycidyl-Bearing Polymers with Biorelevant Sulfhydryl Molecules: Toward Chemoselective Bioconjugation Strategies
Source: Biomacromolecules. 2025 Jul 17;26(8):5269–86. doi: 10.1021/acs.biomac.5c00766 (PMC12344704; doi:10.1021/acs.biomac.5c00766)
Supplement: Supplementary file 1 [file bm5c00766_si_001.pdf]

Supporting Information

TCEP-enabled click modification of glycidyl-bearing polymers with biorelevant sulfhydryl molecules: toward chemoselective bioconjugation strategies.

*Ilaria Porello, Federico Stucchi, Rosachiara Guarini, Giulia Sbaruffati, Francesco Cellesi**

Department of Chemistry, Materials and Chemical Engineering "G. Natta", Politecnico di Milano, Via Luigi Mancinelli 7, 20131, Milan, Italy.

*Corresponding author.
Email address: francesco.cellesi@polimi.it (F. Cellesi).

Materials and Methods.

Synthesis of PCL_r.

The monomer (M) ε-caprolactone (5 g, 43.8 mmol), was inserted in a round double neck flask and three cycles of nitrogen/vacuum of 5 min each were performed. The initiator (I) (eq. M/eq. I = r, where r = 30) and the catalyst (C) $Sn(Oct)_2$ (eq. C/eq. I = 0.1) were then added, maintaining the system under N_2 flow. The mixture was stirred overnight at 130 °C. The reaction was stopped by cooling the flask to room temperature. Purification was carried out by dissolving the crude in DCM (2.5 g/mL) and dropping it into cold MeOH (MeOH/DCM = 100/1 v/v). The system was kept under vigorous stirring in an ice bath throughout the precipitation process. The resulting precipitate was isolated from the supernatant through filtration, and the filtrate was dried under reduced pressure at 50 °C, obtaining the final product as a white powder. Yield > 90%. Conversion χ = 99% (from ^{1}H-NMR data). ^{1}H-NMR (400 MHz, $CDCl_3$), δ (ppm): δ 7.34-7.32 (s, 5H, C_6H_5-CH_2-), 5.11-5.09 (s, 2H, C_6H_5-CH_2-OC(O)-), 4.06-4.03 (t, 2H·(*r*-1), -CH_2-OC(O)-), 3.64-3.61 (t, 2H, -CH_2-OH), 2.30-2.28 (t, 2H·*r*, -OC(O)-CH_2-), 1.64 (m, 4H·*r*, -OC(O)-CH_2CH_2-, -CH_2CH_2-OC(O)-), 1.38-1.36 (m, 2H·*r*, -$CH_2CH_2CH_2$-), where *r* is PCL degree of polymerization.

Synthesis of PCL_r-Br.

5 g of PCL_r (1 eq.) were added in a two-neck flask then evacuated and backfilled with nitrogen three times, dissolved in 6 mL of dry toluene, and cooled at 0 °C in an ice bath. TEA (1.7 eq.) and BiBB (1.5 eq.) were dissolved in 1 mL of toluene each and stirred for 10 min. TEA and BiBB solutions were added dropwise to the reaction mixture through a dropping funnel. The reaction mixture was then brought back to r.t. and stirred overnight at 60 °C in an oil bath. The resulting solution was dried under reduced pressure until toluene complete evaporation, and the crude product was dissolved in 5 mL of DCM. The solution was the filtered to remove the salts formed during the reaction, washing the glassware and the paper filter used with 10 additional mL of DCM to avoid product losses. An aqueous solution (φ_{acq}) of $NaHCO_3$ at pH ≥ 8 was prepared dissolving ~ 3 g of salt in 60 mL of water. The polymer solution (φ_{org}) obtained after the filtration was inserted in a separating funnel and washed three times with the $NaHCO_3$ solution ($\varphi_{org}/\varphi_{acq}$ ~ 1/1 v/v for each wash) to extract the desired product in the organic phase. The water phase collected at the end of the washing steps was mixed with additional DCM ($\varphi_{org}/\varphi_{acq}$ ~ 1/1 v/v) for the complete recovery of the product. Anhydrous Na_2SO_4 was added to the organic solution for water adsorption, with consequent salt removal through filtration. The filtrate was dried under reduced pressure, then dissolved in 2 mL of DCM and dropped in 250 mL of cold MeOH, maintaining the system under stirring in an ice bath. The solution was then

filtered to isolate the precipitate, obtaining the final product as a slightly yellow viscous oil. Yield > 90%. Conversion χ = 100% (from ^{1}H-NMR data). ^{1}H-NMR (400 MHz, $CDCl_3$), δ (ppm): δ 4.06-4.05 (t, 2H·*r*, -CH_2-OC(O)-), 2.30 (t, 2H·*r*, -OC(O)-CH_2-), 1.93-1.92 (s, 6H, -C$(CH_3)_2$Br), 1.65 (m, 4H·*r*, -OC(O)-CH_2CH_2-, -CH_2CH_2-OC(O)-), 1.38 (m, 2H·*r*, -$CH_2CH_2CH_2$-), where *r* is PCL degree of polymerization.

Synthesis of $PGMA_n$.

THF (inhibitor free) was degassed under nitrogen for 30 min. The reaction initiator (I) EBiB (150 mg, 1 eq.) and the monomer GMA (eq. GMA/eq. I = n, n = 30) were added to a Schlenk flask and three cycles of nitrogen/vacuum of 5 min each were performed. The catalyst-ligand solution (C-L) was prepared as it follows: Cu(I)Br (574 mg) and BPY (1148 mg) were inserted in a double neck flask, and three cycles vacuum/nitrogen were performed. Degassed THF (5 mL) was added, obtaining a green mixture that was stirred at r.t. under N_2 for 10 min. THF was added to the reaction mixture ([GMA] ≤ 1 M), and the flask was heated up to 50 °C, until complete dissolution of the solid component. Finally, the required amount of catalyst solution ([C]/[L]/[I] = 1/2/1) was added to the monomers. The reaction mixture was stirred overnight at 50 °C. The purification was performed by filtering the reaction mixture through a neutral alumina pad (h = 3 cm, Φ = 2 cm), washing with THF. The filtrate was dried under reduced pressure and the resulting crude was dissolved in DCM (2 mg/mL) and dropped in cold Et_2O (DCM/Et_2O = 1/100 v/v), maintaining the system under stirring in an ice bath. The mixture was stored at −20 °C for 30 min and the resulting precipitate was isolated by removing the supernatant through filtration.

Synthesis of PCL_r-b-$PGMA_n$.

THF (inhibitor free) was degassed under nitrogen for 30 min. The reaction macroinitiator (I) PCL_r-Br (500 mg, 1 eq.) and the monomer GMA (eq. GMA/eq. I = n, n = 30) were added to a Schlenk flask and three cycles of nitrogen/vacuum of 5 min each were performed. The catalyst-ligand solution (C-L) was prepared as it follows: Cu(I)Br (574 mg) and BPY (1148 mg) were inserted in a double neck flask, and three cycles vacuum/nitrogen were performed. Degassed THF (5 mL) was added, obtaining a green mixture that was stirred at r.t. under N_2 for 10 min. THF was added to the reaction mixture ([GMA] ≤ 1 M), and the flask was heated up to 50 °C, until complete dissolution of the solid component. Finally, the required amount of catalyst solution ([C]/[L]/[I] = 1/2/1) was added to the monomers. The reaction mixture was stirred overnight at 50 °C. The purification was performed by filtering the reaction mixture through a neutral alumina pad (h = 3 cm, Φ = 2 cm), washing with THF. The filtrate was dried under reduced pressure and the resulting crude was dissolved in DCM (2

mg/mL) and dropped in cold Et_2O (DCM/Et_2O = 1/100 v/v), maintaining the system under stirring in an ice bath. The mixture was stored at −20 °C for 30 min and the resulting precipitate was isolated by removing the supernatant through filtration.

Synthesis of PCL_r-b-[$P(PEGMA)_m$-co-$PGMA_n$].

THF (inhibitor free) was degassed under nitrogen for 30 min. The reaction macroinitiator (I) PCL_r-Br (500 mg, 1 eq.) and the monomers PEGMA and GMA were added to a Schlenk flask and three cycles of nitrogen/vacuum of 5 min each were performed. The monomers amount was calculated according to the target degree of polymerization (eq. PEGMA/eq. I = m, eq. GMA/eq. I = n, where m = 30 and n = 3, 4, 5). The catalyst solution (C) was prepared as follows: Cu(I)Br (574 mg) was inserted in a double neck flask, and three cycles vacuum/nitrogen were performed. Degassed THF (5 mL) and the ligand (L) HMTETA (1 mL) were added, obtaining a mixture that was stirred at r.t. under N_2 for 10 min. THF was added to the reaction mixture ([PEGMA] ≤ 1 M), and the flask was heated and kept at 50 °C until complete dissolution of the solid components. Finally, the required amount of catalyst solution ([C]/[L]/[I] = 1/1/1) was added to the monomers. The reaction mixture was stirred overnight at 50 °C. The purification was performed by filtering the reaction mixture through a neutral alumina pad (h = 3 cm, Φ = 2 cm), washing with THF. Then the filtrate was dried under reduced pressure and the resulting crude was dissolved in DCM (2 mg/mL) and dropped in cold Et_2O (DCM/Et_2O = 1/100 v/v), maintaining the system under stirring in ice bath. The mixture was stored at −20 °C for 30 min and the resulting precipitate was isolated by removing the supernatant through filtration.

Introduction

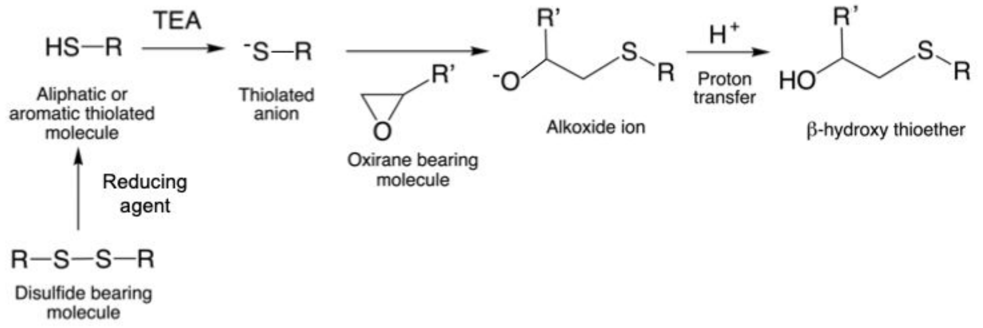

Figure S1. Reaction pathway for general oxirane ring opening process by means of thiolated molecules[61].

Results and Discussion

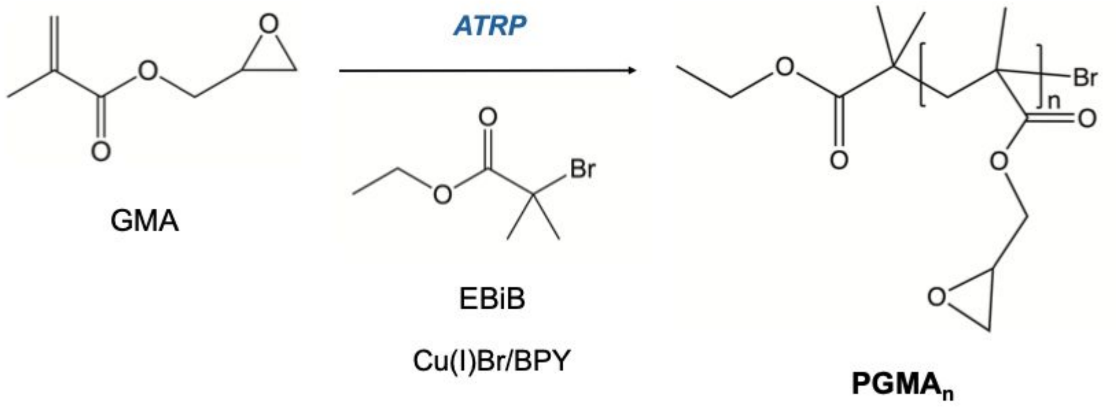

Figure S2. Reaction pathway for $PGMA_n$ homopolymer synthesis via ATRP.

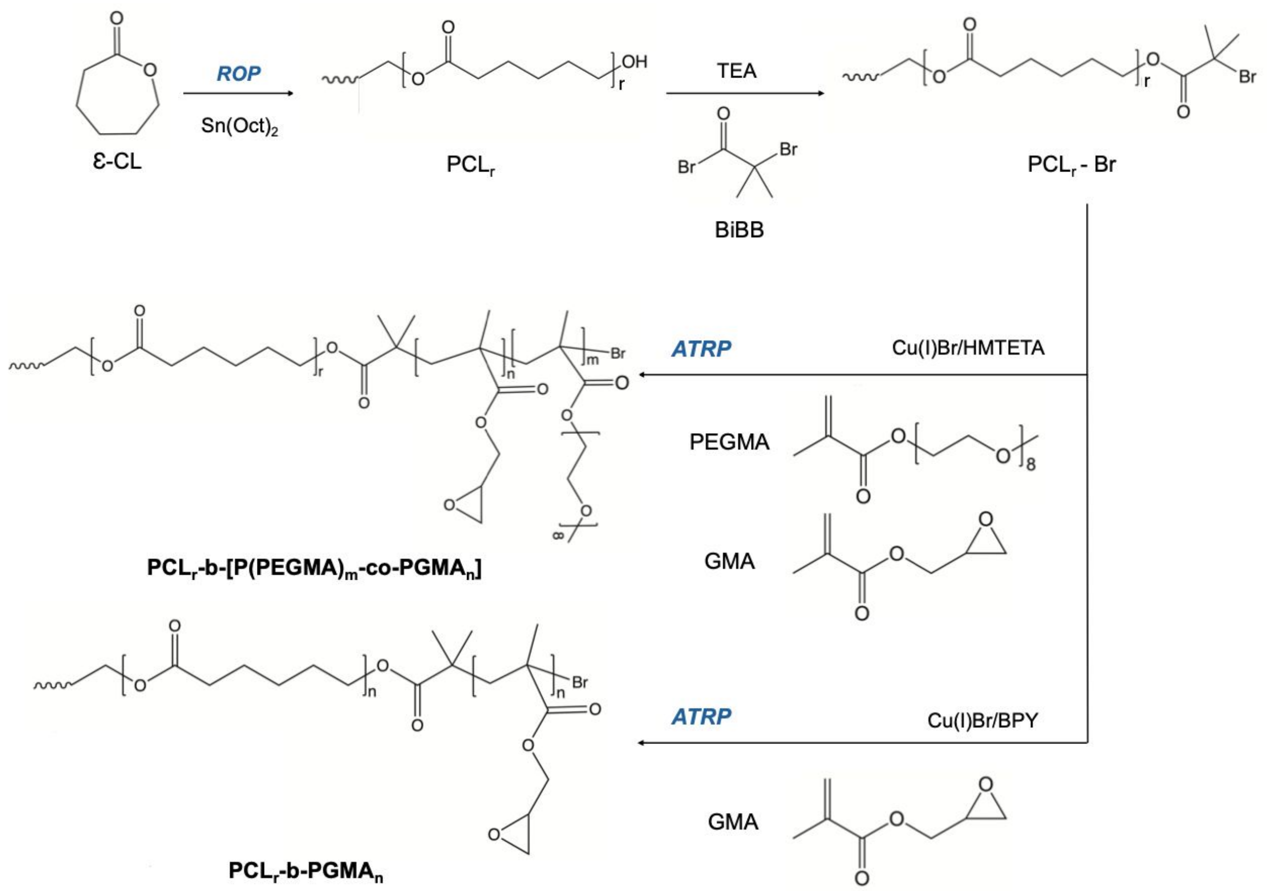

Figure S3. Reaction pathway for PCL_r-b-$PGMA_n$ and PCL_r-b-[$P(PEGMA)_m$-co-$PGMA_n$] copolymers synthesis via ATRP.

a)

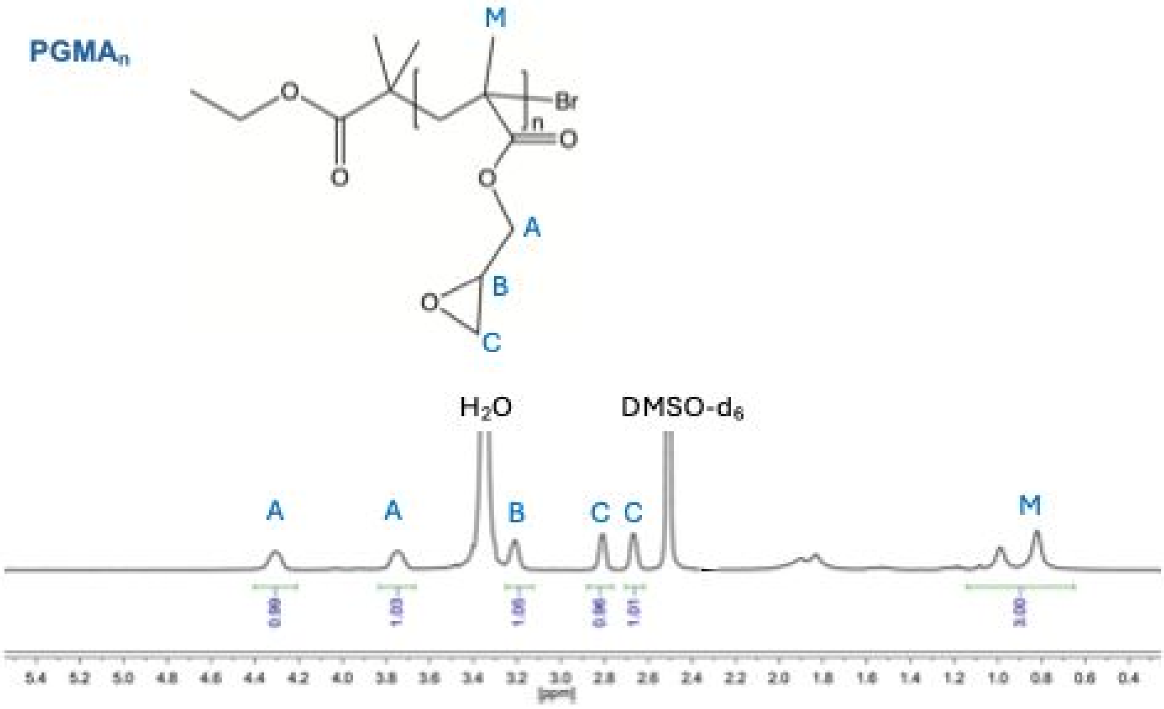

PGMAn
M
Br
n
A
B
C
H2O
DMSO-d6
A
A
B
C C
M
5.4 5.2 5.0 4.8 4.6 4.4 4.2 4.0 3.8 3.6 3.4 3.2 3.0 2.8 2.6 2.4 2.2 2.0 1.8 1.6 1.4 1.2 1.0 0.8 0.6 0.4
[ppm]

b)

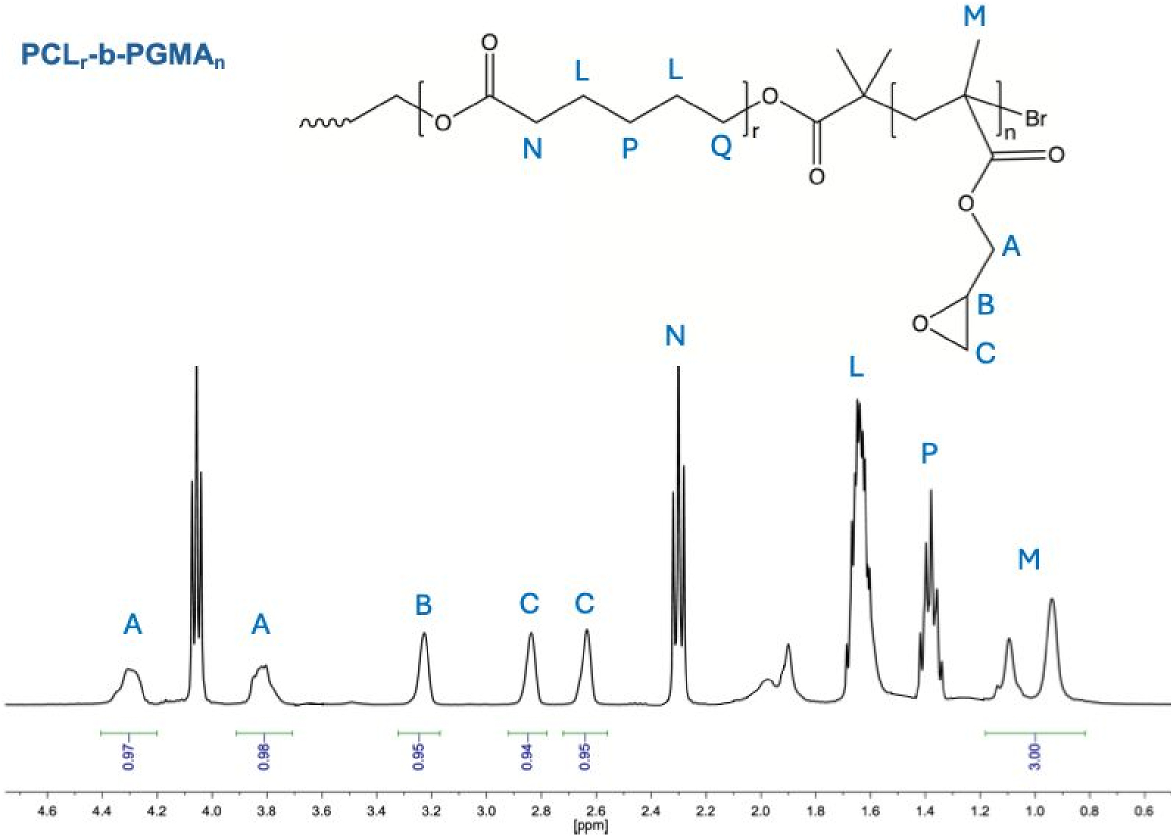

PCLr-b-PGMAn
M
L
L
N
P
Q
r
n
Br
A
B
C
N
L
P
M
A
A
B
C
C
0.97
0.98
0.95
0.94
0.95
3.00
4.6 4.4 4.2 4.0 3.8 3.6 3.4 3.2 3.0 2.8 2.6 2.4 2.2 2.0 1.8 1.6 1.4 1.2 1.0 0.8 0.6
[ppm]

c)

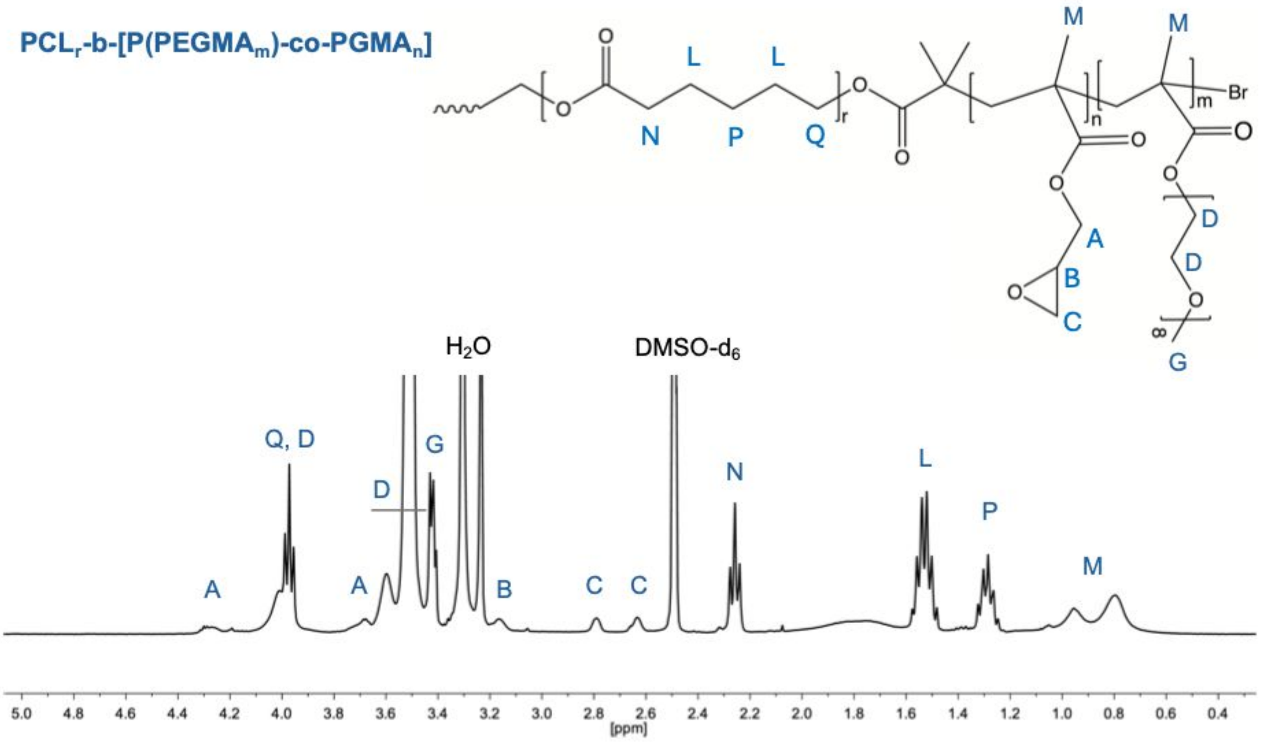

Figure S4. ^{1}H-NMR spectra of **a)** $PGMA_n$ homopolymer (in DMSO-d_6), **b)** PCL_r-b-$PGMA_n$ di-block copolymer (in $CDCl_3$), **c)** PCL_r-b-[P(PEGMA)$_m$-co-$PGMA_n$] block copolymer (in DMSO-d_6).

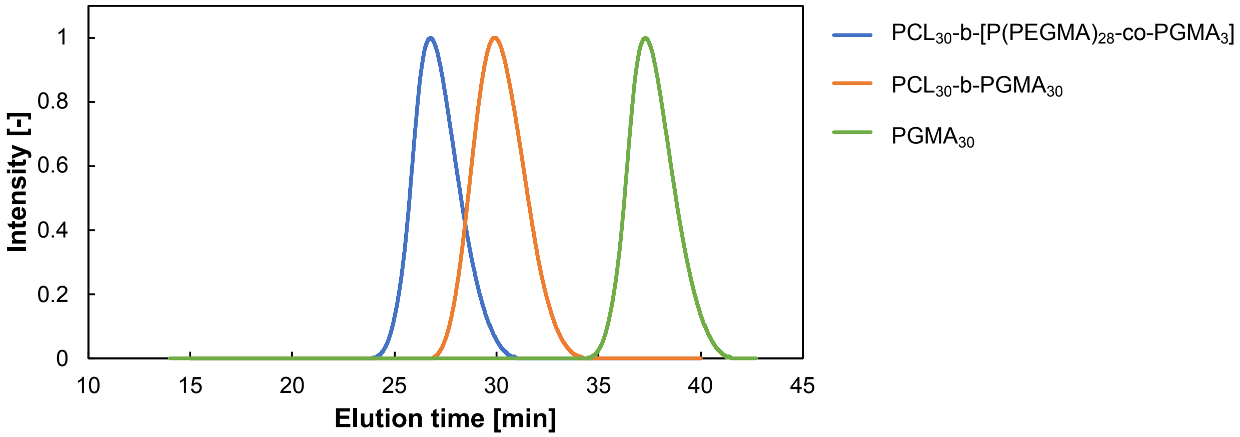

Figure S5. SEC chromatogram (run in THF, 25°C, 1 mL/mim) of **a)** $PGMA_n$ homopolymer, **b)** PCL_r-b-$PGMA_n$ di-block copolymer, **c)** PCL_r-b-[P(PEGMA)$_m$-co-$PGMA_n$] block copolymer.

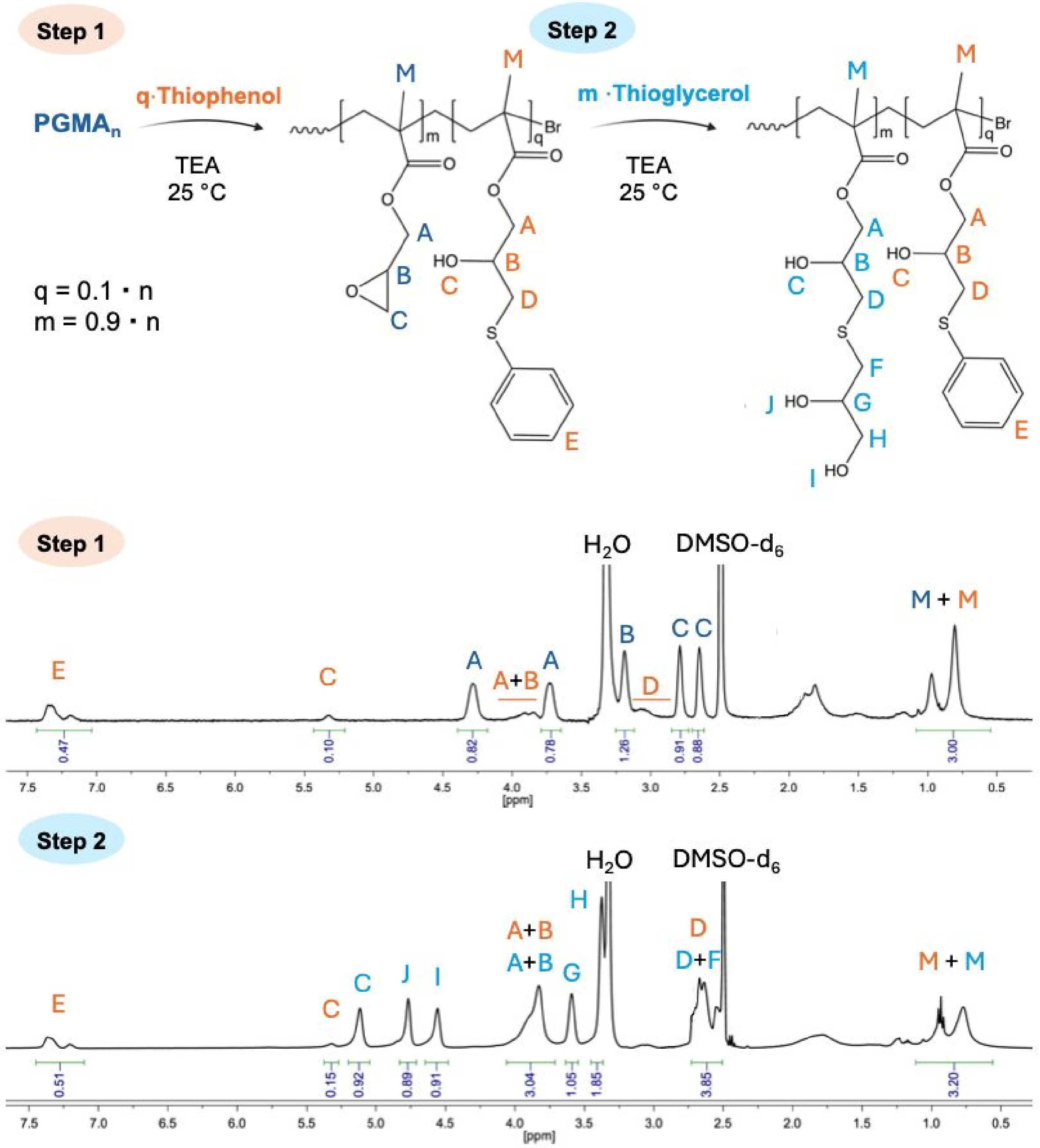

Figure S6. ^{1}H-NMR spectra (in DMSO-d$_6$) recorded after that the 10% of GMA units in a PGMA homopolymer were modified with thiophenol (Step 1) and after complete functionalization via epoxy ring opening with thioglycerol (Step 2). In the first step, the characteristic peak of the thiophenol aromatic ring (7.0 – 7.5 ppm) exhibited an integrated area equal to ≅ 0.5, which was confirmed to be the 10% of the expected value in the case of complete functionalization (corresponding at 5 H$^+$ for each GMA scaffold). Consistently, the peaks of the original PGMA were also reduced by 10%, in parallel with the formation of new emerging peaks. In the second step, the appearance of -OH groups introduced by thioglycerol was clearly visible (4.4 – 5.4 ppm). The epoxide ring signals (2.5 – 3.0 ppm*,* PGMA$_n$ homopolymer spectrum) completely disappeared, while the peaks corresponding to the thiophenol aromatic ring remained unchanged.

a)

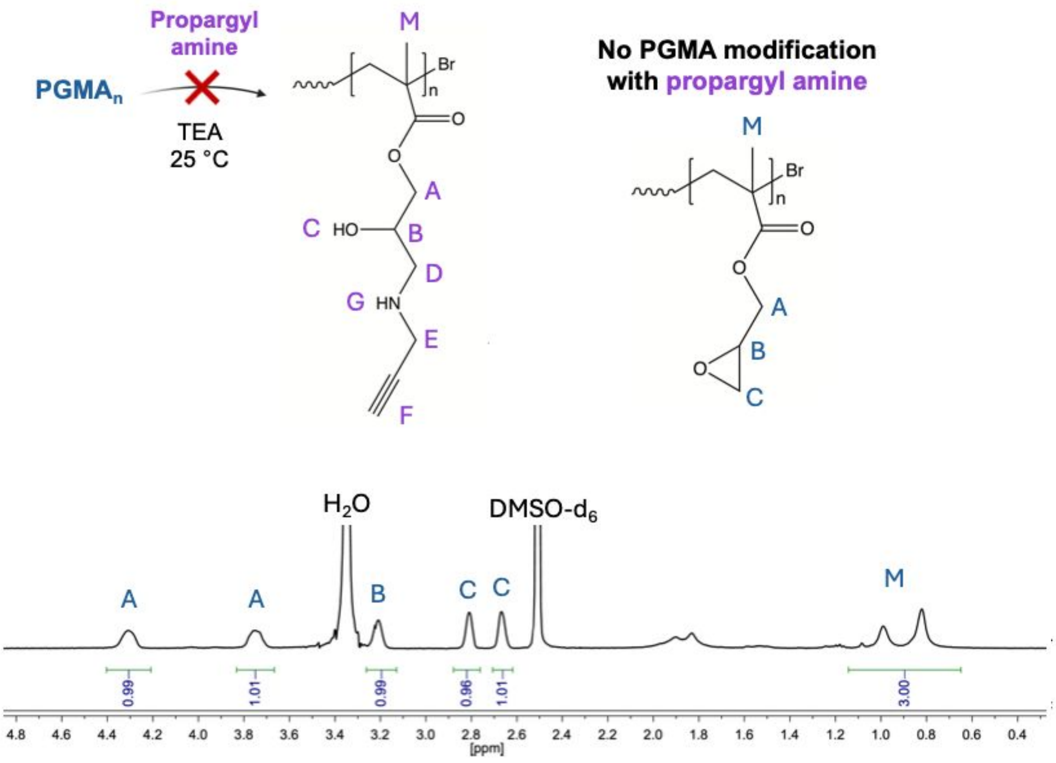

b)

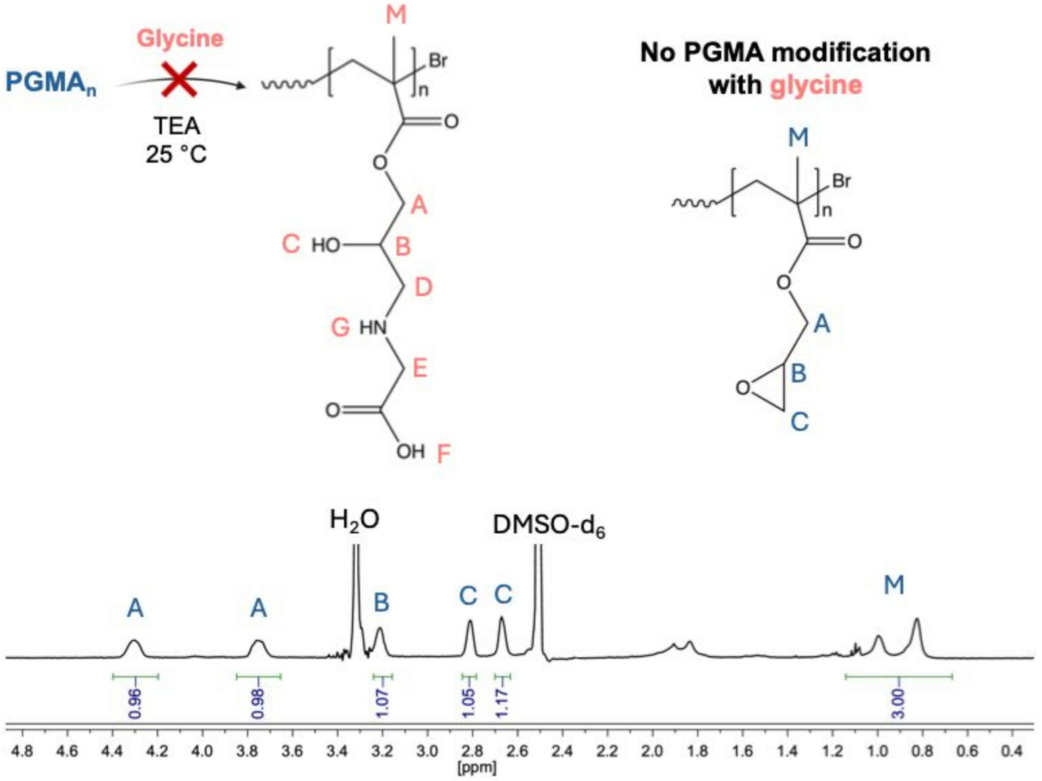

Figure S7. ^{1}H-NMR spectra (in DMSO-d$_6$) of PGMA$_n$ reacted with: **a)** propargyl amine in presence of TEA at 25°C (epoxy ring/propargylamine/TEA = 1/1/3), **b)** glycine amino acid in presence of TEA at 25°C (epoxy ring/glycine/TEA = 1/2/3).

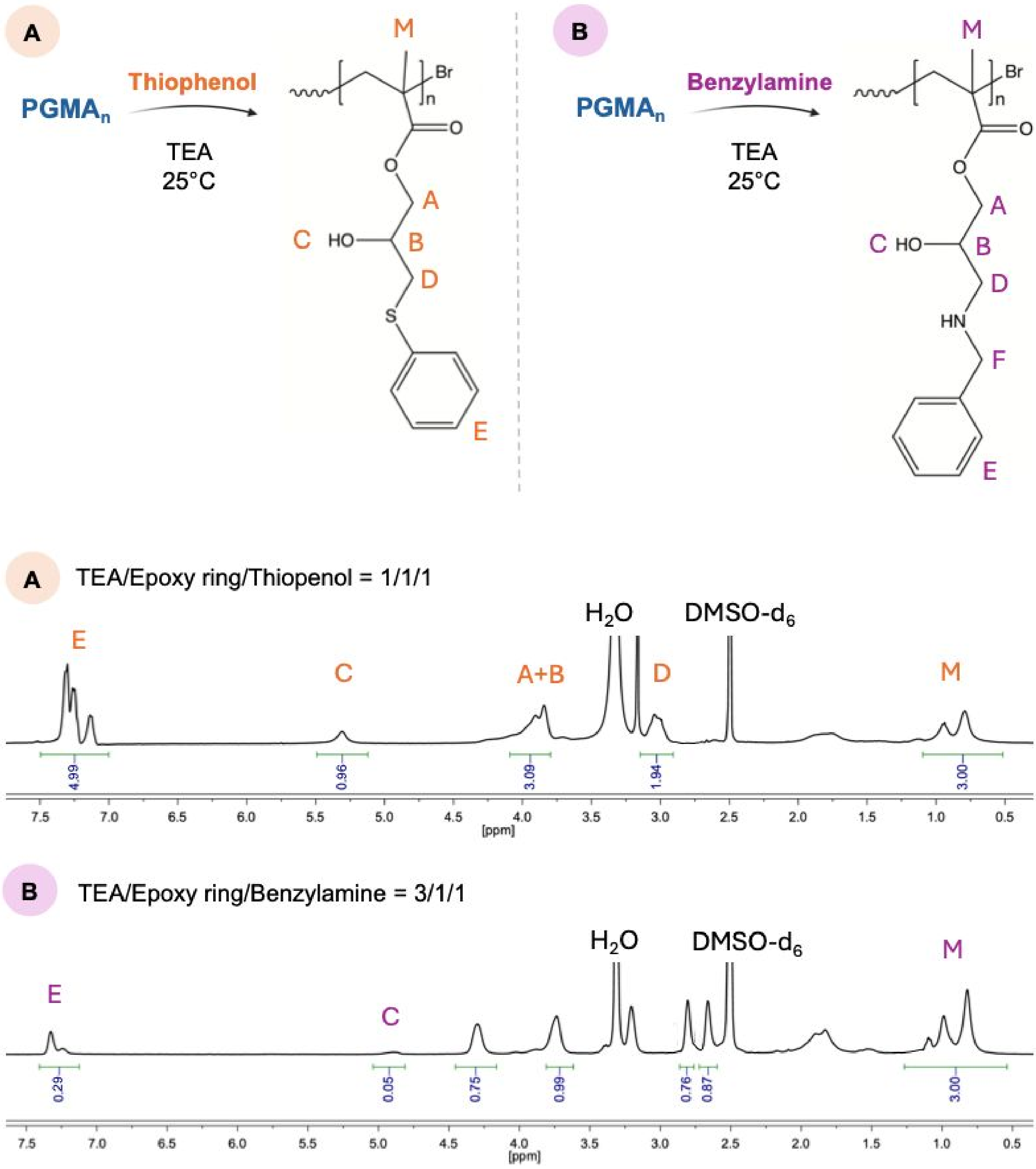

Figure S8. Comparison of ^{1}H-NMR spectra (in DMSO-d_6) recorded after $PGMA_n$ modification with thiophenol (A) and with benzylamine (B). Both the reactions reported were carried out in THF at 25°C, the reactants molar ratios are highlighted in the image. 100% conversion was achieved with thiophenol, while despite the excess of TEA, 5% of functionalization is obtained with benzylamine.

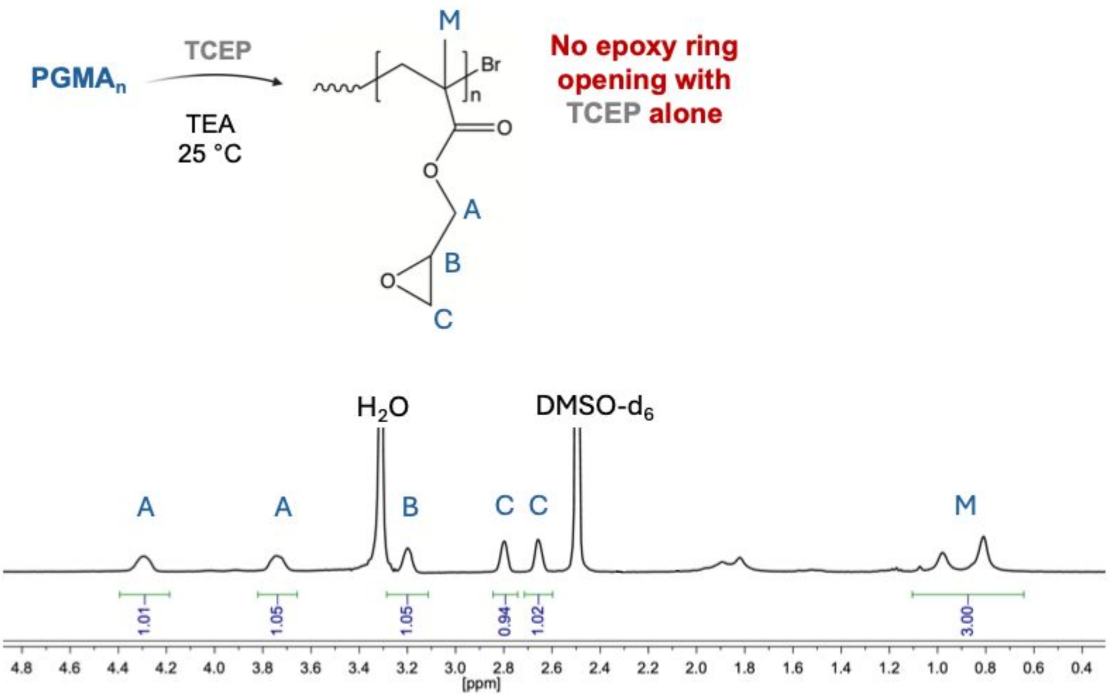

Figure S9. ^{1}H-NMR spectra (in DMSO-d$_6$) recorded after PGMA$_n$ treatment with TCEP (epoxy ring/TCEP = 1/1) in presence of threefold molar excess of TEA at 25° for 18 h.

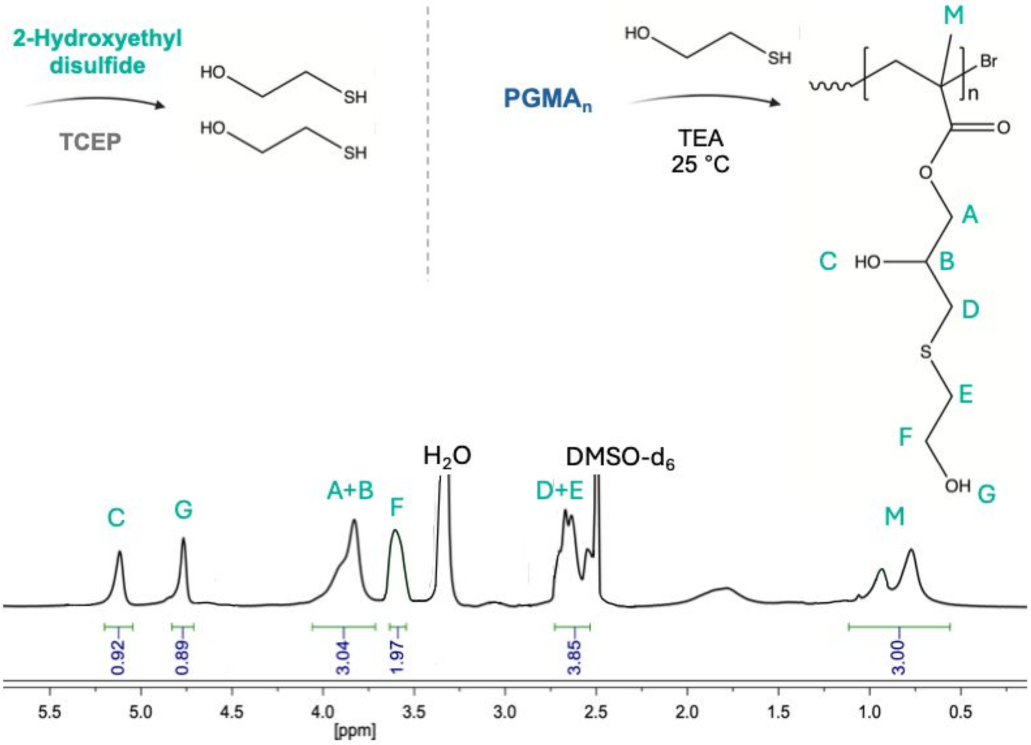

Figure S10. ^{1}H-NMR spectra (in DMSO-d$_6$) of PGMA$_n$ treated with 2-hydroxyethyl disulfide in presence of TEA at 25°C after functionalizing agent -SH groups were activated by means of TCEP.

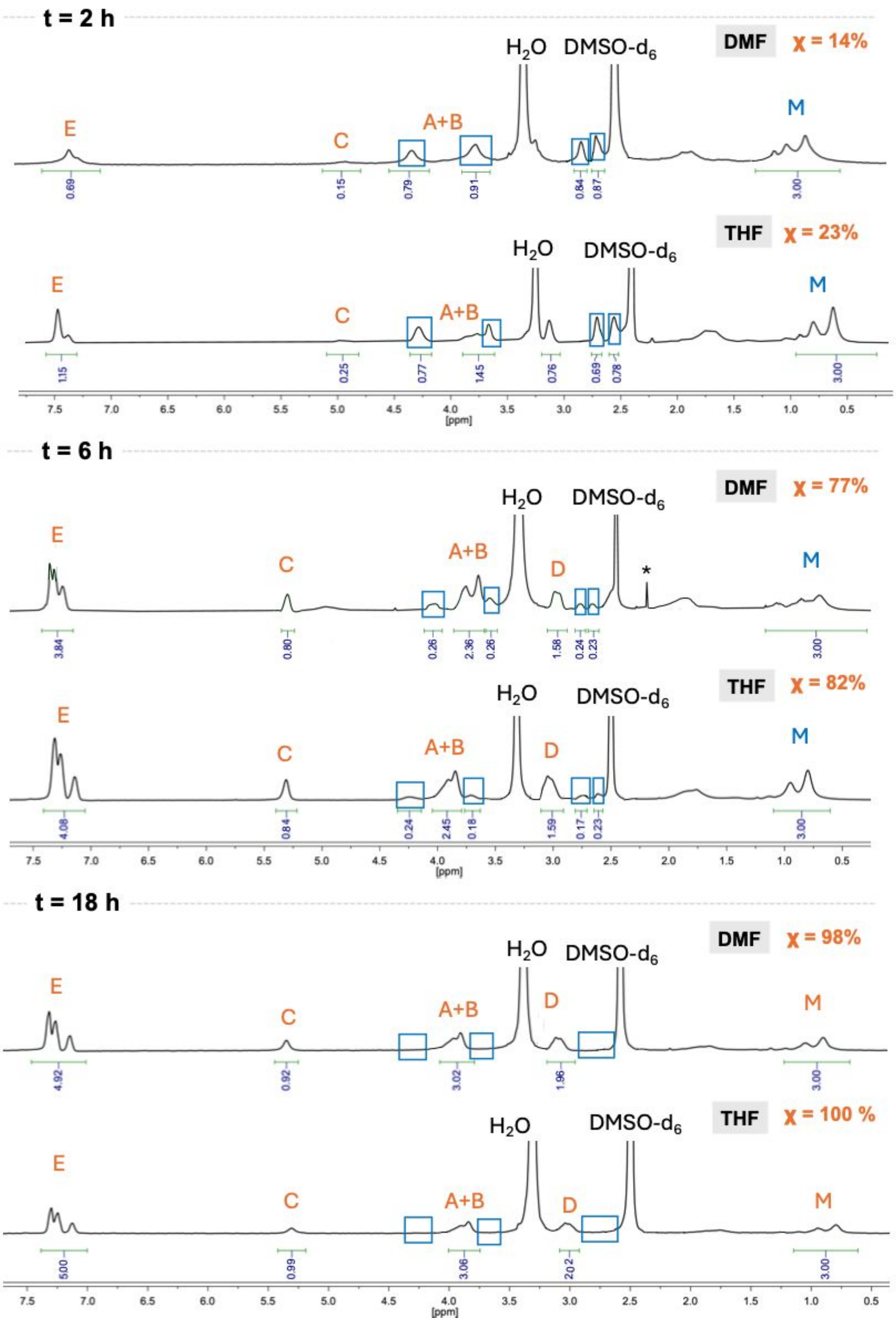

Figure S11. ^{1}H-NMR spectra (in DMSO-d$_6$) of PGMA$_n$ treated with diphenyl disulfide previously reacted with TCEP in presence of TEA at 25°C (epoxy ring/TEA = 1/1) collected at various time points both in THF and DMF.

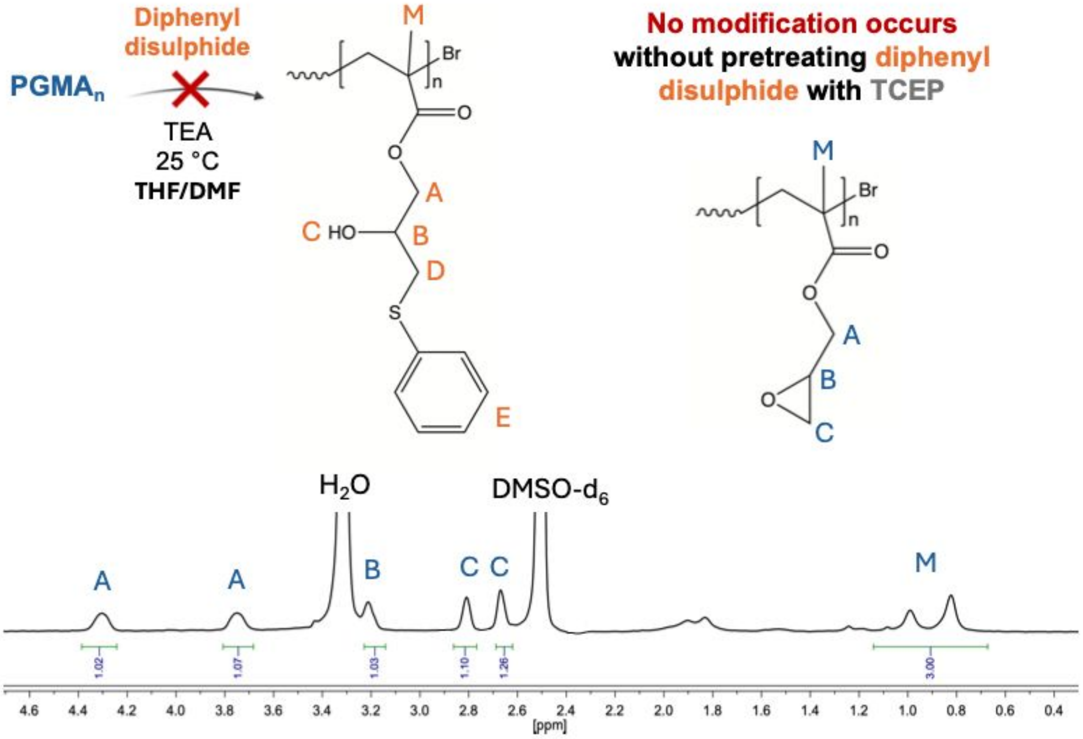

Figure S12. ^{1}H-NMR spectra (in DMSO-d_6) of $PGMA_n$ treated with diphenyl disulfide in presence of TEA at 25°C (epoxy ring/TEA = 1/1) when the functionalizing agent was not formerly treated with TCEP (the same output was obtained both performing the reaction in THF or DMF).

**Polymer mass** *[g]*	0.005
**Volume polymer solution** *[mL]*	10
**GMA units** *[-]*	30
**Polymer $M_{n,NMR}$ after modif. with L-Cys** *[g/mol]*	7899
**n free -SH (attachment from L-Cys NH_2 site)** *[mol]*	6.33E-07 (pol. mass/ M_n after modif.·GMA units)
**DTNB molar extinction coef. E** *[1/M·cm]*	14150
**Path length b** *[cm]*	1
**Volume polymer sol. used for Ellman assay** *[mL]*	0.25
**Volume polymer sol. + DTNB sol.** *[mL]*	2.8
**Abs (412 nm) of polymer sample with DTNB sol.** *[-]*	0.0357
**Conc. free -SH analyzed** *[M]*	3.93E-06 (Abs/E · b)
**n free -SH in polymer sol. used for Ellman assay**	1.10E-08
**n free -SH in polymer sol. tot**	1.10E-07
**n -SH polymer sol. tot/ n -SH polymer (attach. from L-Cys NH_2)**	< 1 %

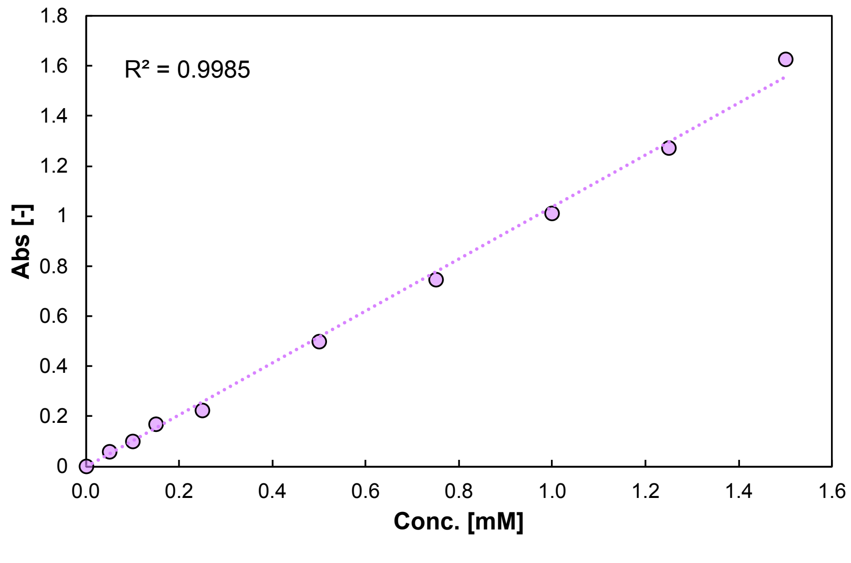

Figure S13. Ellman reagent assay performed on $PGMA_{30}$ modified with L-cysteine after treatment with an immobilized TCEP disulfide reducing gel to detect free -SH. The number of moles of free -SH detected in the analyzed polymer sample was reported to the total number of moles of free -SH that are supposed to be present in case of 100% PGMA modification from NH_2 site of L-cysteine. Calibration curve obtained from pure sample of L-Cys at different concentrations treated with DNTB agent prove that there is a liner correlation between the absorbance detected and the number of free -SH.

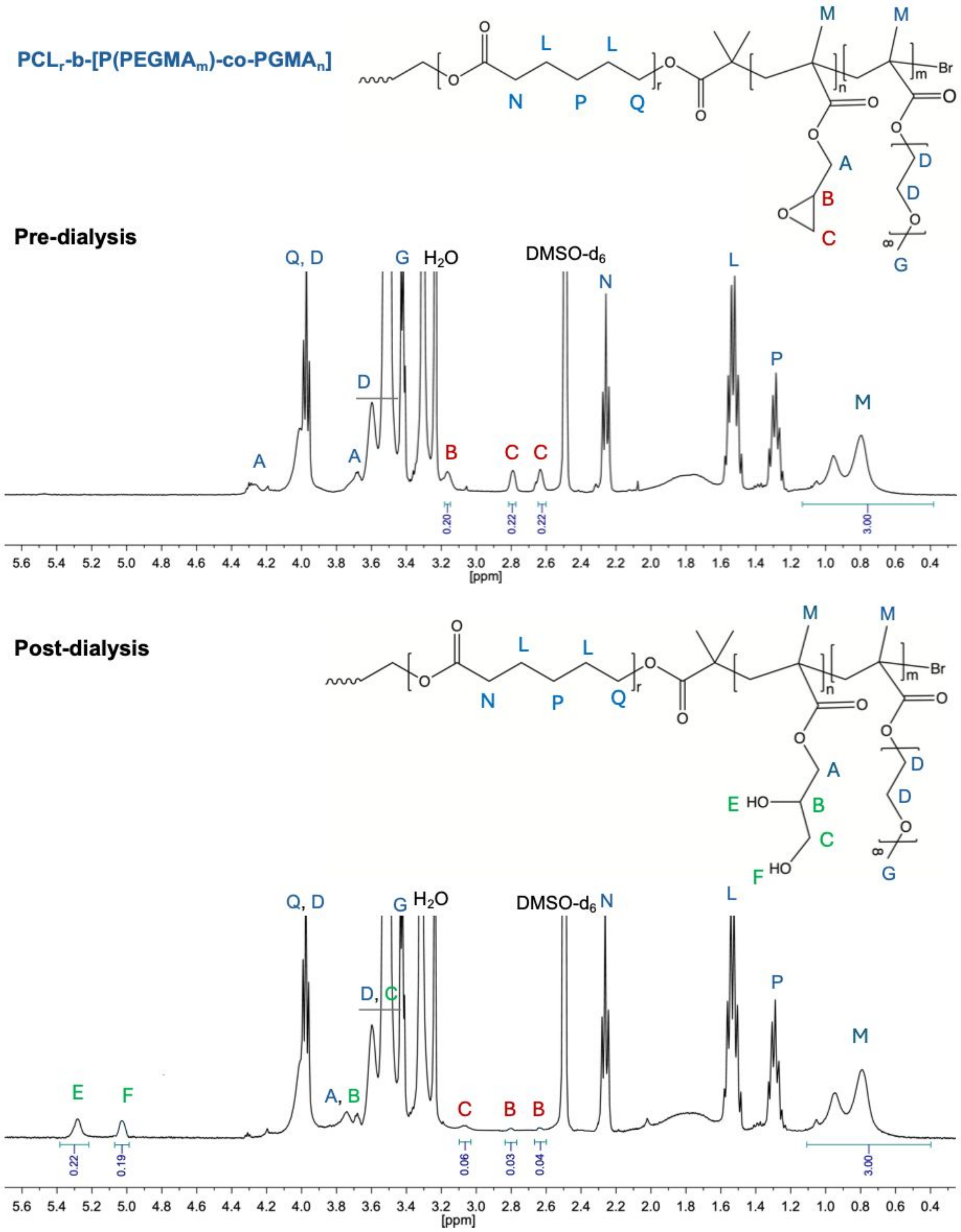

Figure S14. ^{1}H-NMR spectra (in DMSO-d_6) of PCL_r-b-[$P(PEGMA)_m$-co-$PGMA_n$] in its native form (top) and after dialysis against water (bottom) at r.t. for 18 h, highlighting the instability of glycidyl groups in water due to their tendency to undergo ring opening and form diols.

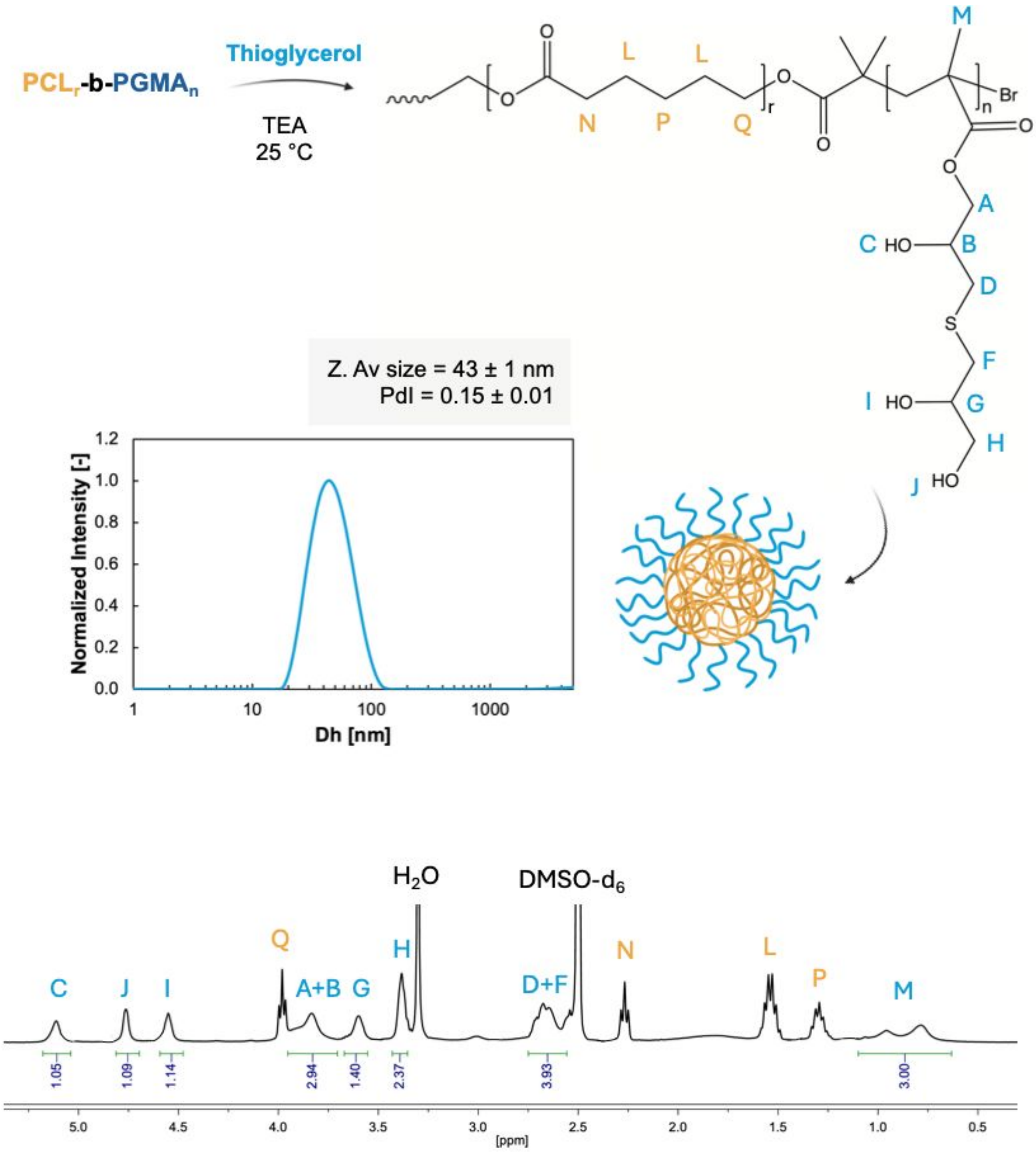

Figure S15. PCL$_r$-b-PGMA$_n$ modification with thioglycerol (epoxy ring/thioglycerol/TEA = 1/1/1) to obtain an amphiphilic molecule able to produce core-shell micelles in aqueous suspension (10 mg/mL in PBS 10 mM pH 7.4). Size distribution curve, Z-Average size and PdI evaluated by DLS analysis are reported as mean values of measurements run in triplicates (top). In the bottom, ^{1}H-NMR spectrum (in DMSO-d$_6$) of the resulting macromolecule.
